# The Role of Endothelin‐1, Kidney Function and Diabetes in Patients With Coronary Artery Disease Underwent Percutaneous Coronary Intervention

**DOI:** 10.1111/1753-0407.70127

**Published:** 2025-07-21

**Authors:** Zixiang Ye, Enmin Xie, Yuan Du, Wenjia Zhang, Kefei Dou

**Affiliations:** ^1^ Department of Cardiology Fuwai Hospital, National Center for Cardiovascular Diseases, Chinese Academy of Medical Sciences and Peking Union Medical College Beijing China; ^2^ State Key Laboratory of Cardiovascular Disease Beijing China; ^3^ Cardiometabolic Medicine Center, Fuwai Hospital, National Center for Cardiovascular Diseases Chinese Academy of Medical Sciences and Peking Union Medical College Beijing China; ^4^ National Clinical Research Center for Cardiovascular Diseases Beijing China

**Keywords:** coronary artery disease, diabetes mellitus, Endothelin‐1, impaired kidney function, major adverse cardiovascular events

## Abstract

**Objective:**

This study aimed to explore the association between plasma big endothelin‐1 (ET‐1) and major adverse cardiovascular events (MACE) in CAD patients who underwent PCI with a focus on the influence of kidney function and diabetes status in secondary prevention.

**Methods:**

A prospective cohort of CAD patients underwent PCI and patients with impaired kidney function and diabetes were initially screened and categorized separately, subdivided based on ET‐1 levels. The primary outcome was MACE, including all‐cause mortality, nonfatal myocardial infarction, unplanned revascularization, and stroke. Statistical analyses included Cox regression, competing risks analysis (competing for non‐cardiovascular death), and restricted cubic spline to assess the relationships between ET‐1 and MACE.

**Results:**

This study included 1344 CAD patients with impaired kidney function and 10,577 CAD patients with DM. During a median follow‐up of 3 years, 20% of renal dysfunction patients and 12.9% of DM patients experienced MACE. In CAD patients with renal dysfunction, elevated ET‐1 levels were associated with increased MACE risk (adjusted HR 1.333, 95% CI 1.169–1.519, *p* < 0.001), with those in the highest group and DM showing a 2.134‐fold (95% CI, 1.334–3.413, *p* < 0.001) increased MACE risk. In CAD patients with DM, patients with eGFR ≤ 60 mL/min/1.73 m^2^ and elevated ET‐1 levels had a 2.297‐fold (95% CI 1.822–2.895) increased risk of MACE.

**Conclusion:**

ET‐1 offered important prognostic value for CAD patients who underwent PCI, with especially bad prognoses observed in those with elevated ET‐1 levels, renal dysfunction, and DM.

## Graphical abstract

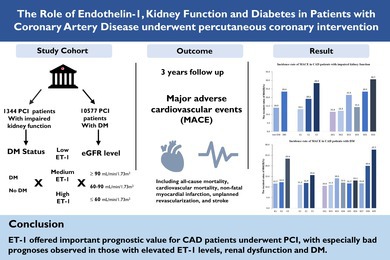

**Summary**

- This study examined the relationship between ET‐1 and MACE in CAD patients with differing renal function and with or without diabetes undergoing PCI.
- CAD Patients who have elevated ET‐1 levels, renal insufficiency (eGFR ≤ 60 mL/min/1.73 m^2^) and DM faced the greatest risk for MACE.
- This study underscored the predictive significance of ET‐1 for MACE in CAD patients who underwent PCI, especially in those with impaired kidney function and DM.

**Abbreviations**
ACS: acute coronary syndrome
AF: atrial fibrillation
AHA: American Heart Association
BMI: body mass index
BNP: brain natriuretic peptide
BUN: blood urea nitrogen
CAD: coronary artery disease
CI: confidence interval
CKD: chronic kidney disease
CMD: coronary microvascular dysfunction
CR: creatinine
CRP: C‐reactive protein
CTO: chronic total occlusion
CV: cardiovascular
DM: diabetes mellitus
eGFR: estimated glomerular filtration rate
ERAs: endothelin receptor antagonists
ET‐1: Endothelin‐1
ETA: endothelin A receptor
ETB: endothelin B receptor
FPG: fasting plasma glucose
HbA1c: glycated hemoglobin
HDL: high‐density lipoprotein
HR: hazard ratio
HTN: hypertension
IQRs: interquartile ranges
LDL: low‐density lipoprotein
MACE: major adverse cardiovascular events
MI: myocardial infarction
PAH: pulmonary arterial hypertension
PCI: percutaneous coronary interventions
ROS: reactive oxygen species
SNP: single nucleotide polymorphism
TC: total cholesterol
TG: triglycerides
TIMI: Thrombolysis in Myocardial Infarction

## 1 Introduction

The prevalence of cardiovascular diseases, such as coronary artery disease (CAD), poses a significant burden on the health system all over the world [1]. In 2023, the American Heart Association (AHA) proposed a novel disease concept—the cardiovascular‐kidney‐metabolic syndrome—highlighting the pathophysiological interactions among metabolism, the kidneys, and the cardiovascular system [2]. The proposal of this concept underscores the importance of coordinated management among the cardiovascular, renal, and metabolic components. Therefore, the shared management of secondary prevention for patients with cardiovascular, renal, and metabolic diseases should be comprehensive and interrelated. However, there has yet to be a systematic and thorough investigation of effective biomarkers that possess significant predictive value across all three domains.

One of the strongest endogenous vasoconstrictors, endothelin‐1 (ET‐1) is linked to a number of illnesses, including liver fibrosis, chronic kidney disease (CKD), hypertension, pulmonary hypertension, and atherosclerosis [3, 4, 5, 6]. According to earlier research, high plasma ET‐1 levels increase the risk of CAD, CKD, and DM [7, 8, 9]. Animal studies have demonstrated that endothelin receptor B (ETB) deficiency prevents adverse effects of metabolic syndrome [10], suggesting it may be a new therapeutic target. However, there is currently no research investigating the possible function of plasma ET‐1 concentration in forecasting unfavorable medical consequences in CAD individuals with various kidney functions and diabetes status undergoing PCI.

Therefore, we proposed a retrospective analysis of a prospective cohort exploring the connection between the plasma ET‐1 levels and poor prognostic outcomes in two study populations including CAD patients undergoing PCI with impaired kidney function in different diabetes statuses and CAD patients undergoing PCI with DM in various kidney functions, respectively.

## 2 Methods

### 2.1 Study Design and Participants

**FIGURE 1:**
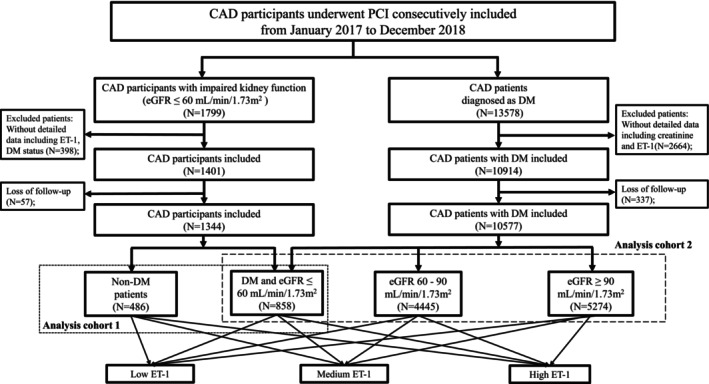
Flowchart of CAD patient underwent PCI selection. ET‐1, Endothelin‐1; CAD, coronary artery disease; eGFR, estimated glomerular filtration rate; DM, diabetes mellitus; PCI, percutaneous coronary intervention.

From January 2017 to December 2018, participants who underwent percutaneous coronary intervention (PCI) at Fuwai Hospital were consecutively enrolled, as illustrated in the flowchart (Figure 1). 1799 CAD participants with impaired kidney function (estimated glomerular filtration rate [eGFR] ≤ 60 mL/min/1.73 m^2^, calculated using CKD‐EPI equations [11]) undergoing PCI were included in the analysis cohort 1, and 13,578 CAD patients with DM were included in the analysis cohort 2, as illustrated in the flowchart. Exclusion criteria included incomplete data (such as baseline ET‐1 and creatinine levels before angiography as well as diabetic status). Ultimately, 1344 CAD patients with impaired kidney function were included and categorized based on diabetes status. 10,577 DM participants were included and divided into three groups according to their baseline eGFR.

The study protocol was approved by the Ethics Committee of Fuwai Hospital, which was carried out in accordance with the Declaration of Helsinki's tenets (Approval Number 2016–847). All participants provided written, informed consent.

### 2.2 Clinical Status and Laboratory Techniques

The decision to perform PCI treatment and all PCI procedures was performed by skilled interventional cardiologists following established standardized guidelines and clinical considerations. Information regarding other comorbid conditions, family history, and previous treatments was obtained through self‐reporting or hospital medical records.

Glycated hemoglobin (HbA1c) readings of ≥ 6.5%, fasting plasma glucose (FPG) levels of ≥ 7.0 mmol/L, or current usage of insulin or antihyperglycemic medications were used to diagnose diabetes. All blood samples were collected prior to coronary angiography and represented the first specimen obtained at admission, before any urgent administration of antiplatelet drugs, heparin, or other medications. A commercially available enzyme immunoassay (BI‐20082H, Biomedica, Vienna, Austria) with a detection sensitivity of 0.02 pmol/L and a normal range of less than 0.25 pmol/L was used to measure the levels of circulating plasma big ET‐1. Plasma glucose was determined using an enzyme‐linked hexokinase assay, while HbA1c levels were assessed using an automated hemoglobin A1c analyzer (HLC‐723G8, Tosoh, Tokyo, Japan).

### 2.3 Clinical Outcomes and Follow‐Up

The study's main endpoint was major adverse cardiovascular events (MACE), which is a composite of all‐cause mortality, cardiovascular mortality, non‐fatal myocardial infarction, unplanned revascularization, and stroke. Death was classified as cardiac unless a clear non‐cardiac explanation could be provided. Positive cardiac troponin levels along with typical chest pain, distinctive electrocardiogram abnormalities, angiographic evidence of intracoronary thrombus, or imaging evidence of acute myocardial necrosis or regional wall motion abnormalities were considered indicators of a non‐fatal myocardial infarction. Neuroimaging investigations and thorough medical histories were used to diagnose stroke. Unplanned revascularization referred to any repeat PCI or surgical intervention occurring after hospital discharge, thus excluding scheduled staged PCI instances.

Patients were followed up every 6 months by trained investigators after discharge. Adverse event data were collected from medical records, clinical visits, and telephone interviews. Two qualified investigators, not involved in the study, independently assessed events using predefined criteria and a centralized review. For events with discrepancies, reassessment was performed by the third investigator.

### 2.4 Statistical Analysis

Categorical data were displayed as frequencies and percentages, whereas baseline characteristics for continuous variables were represented as medians with interquartile ranges (IQRs). The chi‐square test was employed for categorical variables, while the Wilcoxon rank‐sum test, Kruskal‐Wallis test, or analysis of variance (ANOVA) were used to assess the statistical significance of differences for continuous data.

Because ET‐1 pathways may be potentially affected in patients with renal impairment or DM, a more rigorous analysis was performed by setting different cutoff values for the two groups. Participants were split into three groups based on the tertiles of plasma ET‐1 concentrations: low, intermediate, and high. The threshold values were 0.28 pmol/L and 0.44 pmol/L in individuals with impaired renal function, and 0.21 pmol/L and 0.33 pmol/L in those with DM. Kaplan–Meier survival analysis was employed to compare cumulative hazard rates. The relationships between ET‐1 levels, diabetic status, kidney functions, and poor prognosis were analyzed through Cox regression analyses. The Cox proportional hazards assumption was tested via Schoenfeld Residuals. Model 1 was unadjusted, while Model 2 was a fully adjusted model for confounding factors including age, sex, body mass index (BMI), health status (acute coronary syndrome[ACS], smoking status, hypertension, dyslipidemia, family history of CAD), brain natriuretic peptide levels, medications at discharge (statins, aspirin, clopidogrel, ticagrelor, angiotensin II receptor blockers/angiotensin‐converting enzyme inhibitors, beta‐blockers, and calcium channel blockers), and angiographic characteristics (heavily calcified lesions, Syntax score, lesion length, chronic total occlusion, bifurcation, stent usage). A Fine‐Gray subdistribution hazard model adjusted for competing risk as sensitivity analysis was conducted (Fine‐Gray analysis for non‐cardiac death as competing risk). Restricted cubic spline analysis was conducted to evaluate the presence of linear relationships between ET‐1 and various adverse outcomes across different diabetes statuses and kidney functions.

A two‐sided *p*‐value of < 0.05 was considered statistically significant. All statistical analyses were performed using STATA version 17.0 and R version 4.1.3.

## 3 Result

### 3.1 Baseline Characteristics

A total of 1344 CAD patients with renal dysfunction were included in this study; their median age was 69.8 years, and 59.4% of them were male. The baseline characteristics are summarized in Table 1 and Table S1. The median level of ET‐1 was 0.35 pmol/L, with a mean of 0.47 pmol/L. Based on their ET‐1 levels, patients were divided into three groups: low, intermediate, and high, with cut‐off values of 0.28 and 0.44 pmol/L. Age, BNP, FPG, HbA1c, C‐reactive protein (CRP), and creatinine levels all increased with rising plasma ET‐1 concentrations. Additionally, the occurrence rates of ACS, atrial fibrillation (AF), and DM among CAD patients with poor kidney function also rose with increasing ET‐1 levels. Conversely, left ventricular ejection fraction (LVEF), HDL, hemoglobin (HBG), and eGFR decreased as ET‐1 levels increased. Depending on whether they had diabetes or not, the patients were split into two groups: those with diabetes and those without (Table S2). Notably, the ET‐1 levels in diabetic patients were significantly higher than those in non‐diabetic patients.

**TABLE 1:** Baseline characteristics of CAD patients with impaired kidney function in various ET‐1 level.

	Overall	Low ET‐1	Intermediate ET‐1	High ET‐1	*p*
*n*	1344	465	449	430	
Male (%)	799 (59.4)	283 (60.9)	277 (61.7)	239 (55.6)	0.136
Age, y	69.80 [63.20, 75.90]	68.10 [61.90, 73.50]	70.20 [63.40, 76.50]	70.95 [64.62, 77.20]	< 0.001
BMI, kg/m^2^	25.69 [23.53, 27.70]	25.71 [23.66, 27.68]	25.65 [23.63, 27.72]	25.60 [23.35, 27.88]	0.88
Clinical presentation					
ACS (%)	924 (68.8)	305 (65.6)	296 (65.9)	323 (75.1)	0.003
Atrial fibrillation (%)	120 (8.9)	21 (4.5)	35 (7.8)	64 (14.9)	< 0.001
DM (%)	858 (63.8)	258 (55.5)	277 (61.7)	323 (75.1)	< 0.001
Dyslipidemia (%)	1049 (78.1)	372 (80.0)	353 (78.6)	324 (75.3)	0.229
HTN (%)	1105 (82.2)	384 (82.6)	374 (83.3)	347 (80.7)	0.583
Family history CAD (%)	137 (10.2)	46 (9.9)	50 (11.1)	41 (9.5)	0.71
Smoking (%)	705 (52.5)	251 (54.0)	235 (52.3)	219 (50.9)	0.658
Laboratory data					
ET, pmol/L	0.35 [0.24, 0.50]	0.22 [0.18, 0.24]	0.35 [0.32, 0.40]	0.60 [0.51, 0.81]	< 0.001
Glu, mmol/L	6.59 [5.45, 8.75]	6.03 [5.34, 7.91]	6.55 [5.45, 8.50]	7.36 [5.72, 10.12]	< 0.001
HbA1C, %	6.50 [5.90, 7.60]	6.30 [5.80, 7.30]	6.50 [5.90, 7.50]	6.70 [6.00, 7.88]	< 0.001
BUN, mmol/L	7.70 [6.30, 9.30]	7.40 [6.16, 8.97]	7.60 [6.30, 9.10]	8.16 [6.55, 10.04]	< 0.001
eGFR, mL/min/1.73 m2	50.32 (8.80)	52.64 (6.55)	50.53 (7.96)	47.59 (10.79)	< 0.001
Angiographic and PCI data					
Heavily calcified (%)	77 (5.7)	27 (5.8)	28 (6.2)	22 (5.1)	0.772
Syntax	14.00 [7.00, 21.00]	13.00 [7.00, 20.00]	13.00 [7.00, 20.00]	14.75 [8.00, 22.00]	0.162
Triple vessel (%)	750 (55.8)	241 (51.8)	264 (58.8)	245 (57.0)	0.088
MLD, mm	0.30 [0.04, 0.50]	0.30 [0.15, 0.52]	0.30 [0.14, 0.50]	0.25 [0.00, 0.46]	< 0.001
Stent length, mm	28.00 [18.00, 41.00]	28.00 [18.00, 39.00]	30.00 [18.00, 44.00]	26.00 [18.00, 40.00]	0.243
Stent (%)	1196 (89.0)	408 (87.7)	398 (88.6)	390 (90.7)	0.354
Medications at discharge					
Beta (%)	1205 (89.7)	411 (88.4)	402 (89.5)	392 (91.2)	0.393
Statin (%)	1327 (98.7)	463 (99.6)	444 (98.9)	420 (97.7)	0.038
Aspirin (%)	1331 (99.0)	461 (99.1)	443 (98.7)	427 (99.3)	0.6

Abbreviations: ACEI, angiotensin converting enzyme inhibitors; ACS, acute coronary syndrome; ARB, angiotensin II receptor blockers; BMI, body mass index; BNP, brain natriuretic peptide; CCB, calcium channel blockers; CTO, chronic total occlusion; DM, diabetes mellitus; eGFR, estimated glomerular filtration rate; ET‐1; Endothelin‐1; HBG, hemoglobin; HDL‐C, high‐density lipoprotein cholesterol; LDL‐C, low‐density lipoprotein cholesterol; LM, left main coronary artery; LMWH, low molecular weight heparin; MLD, minimal lumen diameter; PCI, percutaneous coronary intervention; PLT, platelet; SYNTAX, synergy between percutaneous coronary intervention with TAXUS and cardiac surgery; TC, total cholesterol; TG, triglycerides; TIMI, Thrombolysis In Myocardial Infarction; WBC, white blood cell.

Table S3 summarized the baseline characteristics of the 10 577 CAD participants with DM in the study (median age: 61.4 years; male: 74.2%). Based on their eGFR, the individuals were divided into three groups as follows: eGFR ≥ 90 mL/min/1.73 m^2^ (5274 subjects, 49.8%), 60 < eGFR < 90 mL/min/1.73 m^2^ (4445 subjects, 42.0%), and eGFR ≤ 60 mL/min/1.73 m^2^ (858 subjects, 8.2%). Notably, plasma levels of BNP, HbA1c, TG, TC, LDL, blood urea nitrogen (BUN), and ET‐1 exhibited a positive correlation with baseline eGFR levels (all *p* < 0.05). Based on their ET‐1 levels, patients were divided into three groups with cut‐off values of 0.21 and 0.33 pmol/L: low, intermediate, and high.

### 3.2 ET‐1 Levels and Clinical Outcomes

**FIGURE 2:**
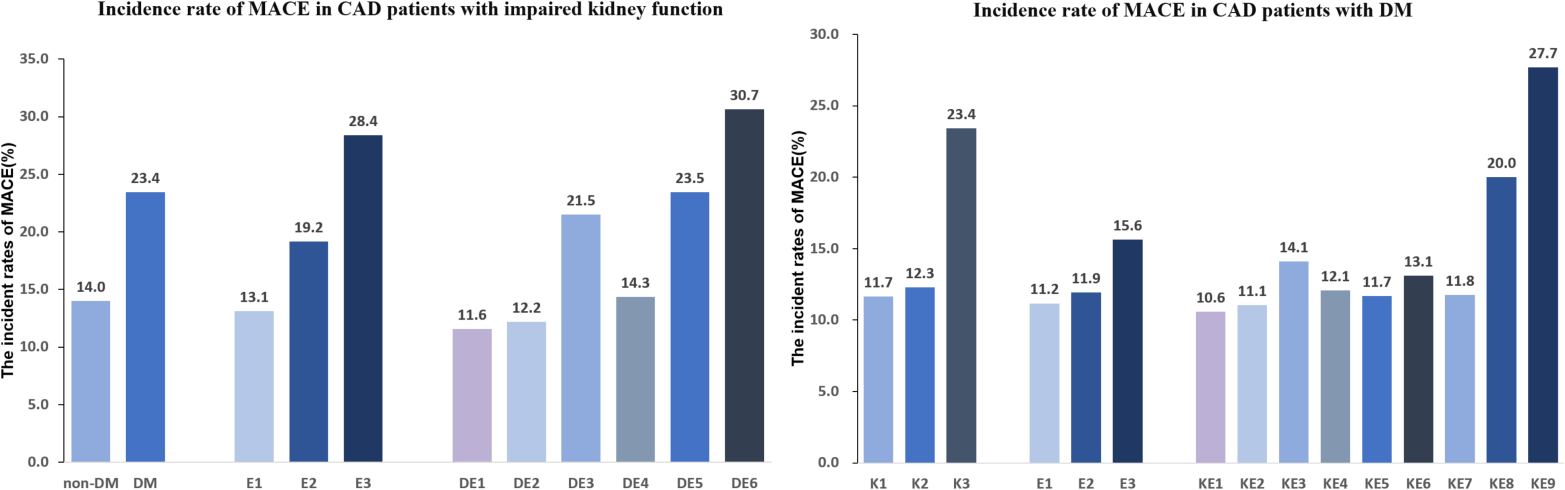
The MACE risks in various CAD groups. (A) Incidence rate of MACE in CAD patients with impaired kidney function; (B) Incidence rate of MACE in CAD patients with DM. E1, low ET‐1 level; E2, medium ET‐1 level; E3, high ET‐1 level; DE1, non‐DM plus low ET‐1 level; DE2, non‐DM plus medium ET‐1 level; DE3, non‐DM plus high ET‐1 level; DE4, DM plus low ET‐1 level; DE5, DM plus medium ET‐1 level; DE6, DM plus high ET‐1 level. K1, eGFR ≥ 90 mL/min/1.73 m^2^; K2, eGFR 60–90 mL/min/1.73 m^2^; K3, eGFR ≤ 60 mL/min/1.73 m^2^; E1, low ET‐1 level; E2, medium ET‐1 level; E3, high ET‐1 level; KE1, eGFR ≥ 90 mL/min/1.73 m^2^ plus low ET‐1 level; KE2, eGFR ≥ 90 mL/min/1.73 m^2^ plus medium ET‐1 level; KE3, eGFR ≥ 90 mL/min/1.73 m^2^ plus high ET‐1 level; KE4, eGFR 60–90 mL/min/1.73 m^2^ plus low ET‐1 level; KE5, eGFR 0–90 mL/min/1.73 m^2^ plus medium ET‐1 level; KE6, eGFR 60–90 mL/min/1.73 m^2^ plus high ET‐1 level; KE7, eGFR ≤ 60 mL/min/1.73 m^2^ plus low ET‐1 level; KE8, eGFR ≤ 60 mL/min/1.73 m^2^ plus medium ET‐1 level; KE9, eGFR ≤ 60 mL/min/1.73 m^2^ plus high ET‐1 level. ET‐1, Endothelin‐1; MACE, major adverse cardiovascular events; DM, diabetes mellitus.

During the follow‐up period (median follow‐up time of 3.12 years, range 2.98 to 3.26 years), CAD patients with impaired kidney function underwent PCI, and 269 MACE occurred (20%), including 129 cases of all‐cause mortality, 56 cases of cardiovascular mortality, 51 cases of non‐fatal MI, 17 strokes, and 124 cases of unplanned revascularization therapy (Figure 2). Participants were also split into three groups according to plasma ET‐1 concentrations with cut‐off values of 0.28 and 0.44 pmol/L.

Univariable and multivariable Cox regression analysis indicated a significant association between elevated ET‐1 levels and increased risk of MACE (Table 2). After full adjustment for relevant covariates, ET‐1 level was positively associated with elevated risks of MACE (adjusted hazard ratio [HR], 1.333; 95% confidence interval [CI], 1.169–1.519, *p* < 0.001). Moreover, high ET‐1 groups demonstrated a 1.695‐fold increased risk of MACE compared with the low ET‐1 group (adjusted HR, 1.694; 95% CI, 1.219–2.353, *p* < 0.001).

**TABLE 2:** Univariate and multivariate Cox regression analyses for various adverse clinical outcomes risk related to ET‐1 levels in CAD patients with impaired kidney function.

MACE	Events	Robust model	Adjusted model
Low ET‐1	61	Reference	Reference
Medium ET‐1	86	1.498 (1.078–2.082)^a^	1.354 (1.068–1.893)^a^
High ET‐1	122	2.303 (1.692–3.134)^a^	1.694 (1.219–2.353)^a^
All‐cause death			
Low ET‐1	16	Reference	Reference
Medium ET‐1	36	2.395 (1.328–4.316)^a^	2.108 (1.161–3.828)^a^
High ET‐1	77	5.490 (3.203–9.409)^a^	3.562 (2.022–6.274)^a^
CV‐cause death			
Low ET‐1	3	Reference	Reference
Medium ET‐1	13	4.565 (1.301–16.019)^a^	4.220 (1.191–14.953)^a^
High ET‐1	40	15.083 (4.664–48.774)^a^	9.664 (2.897–32.237)^a^
Nonfatal myocardial infarction			
Low ET‐1	11	Reference	Reference
Medium ET‐1	12	1.148 (0.504–2.608)	1.041 (0.442–2.451)
High ET‐1	28	2.683 (1.321–5.446)^a^	2.203 (1.018–4.764)^a^
Stroke			
Low ET‐1	5	Reference	Reference
Medium ET‐1	4	0.845 (0.227–3.150)	0.803 (0.206–3.122)
High ET‐1	8	1.845 (0.603–5.643)	1.953 (0.582–6.551)
Revascularization			
Low ET‐1	39	Reference	Reference
Medium ET‐1	46	1.245 (0.810–1.914)	1.174 (0.754–1.827)
High ET‐1	39	1.251 (0.737–1.797)	1.190 (0.612–1.603)

*Note:* Adjusted Model adjusted for age, sex, body mass index (BMI), health status (acute coronary disease, smoking status, hypertension, dyslipidemia, family history of CAD), brain natriuretic peptide levels, medications at discharge (statins, aspirin, clopidogrel, ticagrelor, angiotensin II receptor blockers/angiotensin‐converting enzyme inhibitors, beta‐blockers, and calcium channel blockers), and angiographic characteristics (e.g., heavily calcified lesions, Syntax score, lesion length, chronic total occlusion, bifurcation, stent usage).

Abbreviations: CV, cardiovascular; ET‐1, Endothelin‐1; MACE, major adverse cardiovascular events.

^a^ *P* < 0.05.

Furthermore, patients with elevated ET‐1 levels experienced a markedly higher risk of cardiovascular mortality compared with those with lower ET‐1 levels, consistent with the MACE results. In addition, compared with the reference group, those with elevated ET‐1 levels had a noticeably higher risk of non‐fatal myocardial infarction (adjusted HR, 2.203; 95% CI, 1.018–4.764, *p* < 0.001). However, no significant statistical association was found between ET‐1 levels and stroke or unplanned revascularization (Table 2).

In CAD patients with DM who underwent PCI, 1363 participants experienced MACE, representing 12.9% of the cohort. This included 304 cases of all‐cause mortality, 124 deaths due to cardiovascular causes, 282 nonfatal MI, 985 unplanned revascularizations, and 64 nonfatal strokes. Participants were categorized into three groups based on plasma ET‐1 concentration tertiles: low ET‐1 (≤ 0.21 pmol/L), medium ET‐1 (0.21–0.33 pmol/L), and high ET‐1 (> 0.33 pmol/L).

Elevated ET‐1 levels were linked to a 1.492‐fold increased risk of MACE (HR 1.492; 95% CI 1.311–1.698; *p* < 0.001), according to univariate Cox regression analysis (Table S4). In a multivariate Cox regression model adjusted for additional covariates, ET‐1 was associated with MACE as a continuous variable (adjusted HR 1.149; 95% CI 1.057–1.248; *p* < 0.001). Further, elevated plasma ET‐1 levels were significantly linked to MACE (adjusted HR 1.279; 95% CI 1.118–1.464; *p* < 0.001) compared with the low ET‐1 group.

Elevated plasma ET‐1 was associated with a higher risk of all‐cause mortality (adjusted HR 2.188, 95% CI 1.585–3.022, *p* < 0.001) and cardiovascular mortality (adjusted HR 3.824, 95% CI 2.028–7.210, *p* < 0.001). A positive correlation between plasma ET‐1 levels and the risk of nonfatal MI was also observed (for medium ET‐1: adjusted HR 1.436; 95% CI 1.038–1.987, *p* < 0.001; for high ET‐1: adjusted HR 1.970; 95% CI 1.436–2.702, *p* < 0.001). However, no statistically significant relationship was found between ET‐1 levels and the incidence of strokes or unplanned revascularizations.

### 3.3 Diabetes, ET‐1 Levels, and Clinical Outcomes

Participants with impaired kidney function were further categorized into non‐diabetic (486, 36.1%) and diabetic groups (858, 63.9%). The risk of MACE varied between diabetic and non‐diabetic patients with impaired kidney function, with diabetic patients exhibiting a higher incidence than their non‐diabetic counterparts. Individuals with diabetes had a substantially higher cumulative risk of MACE than individuals without the disease, according to Kaplan–Meier curves. Furthermore, compared with patients with intermediate or low ET‐1 levels, those with high ET‐1 levels had the highest cumulative risk of MACE (Figure S1).

**FIGURE 3:**
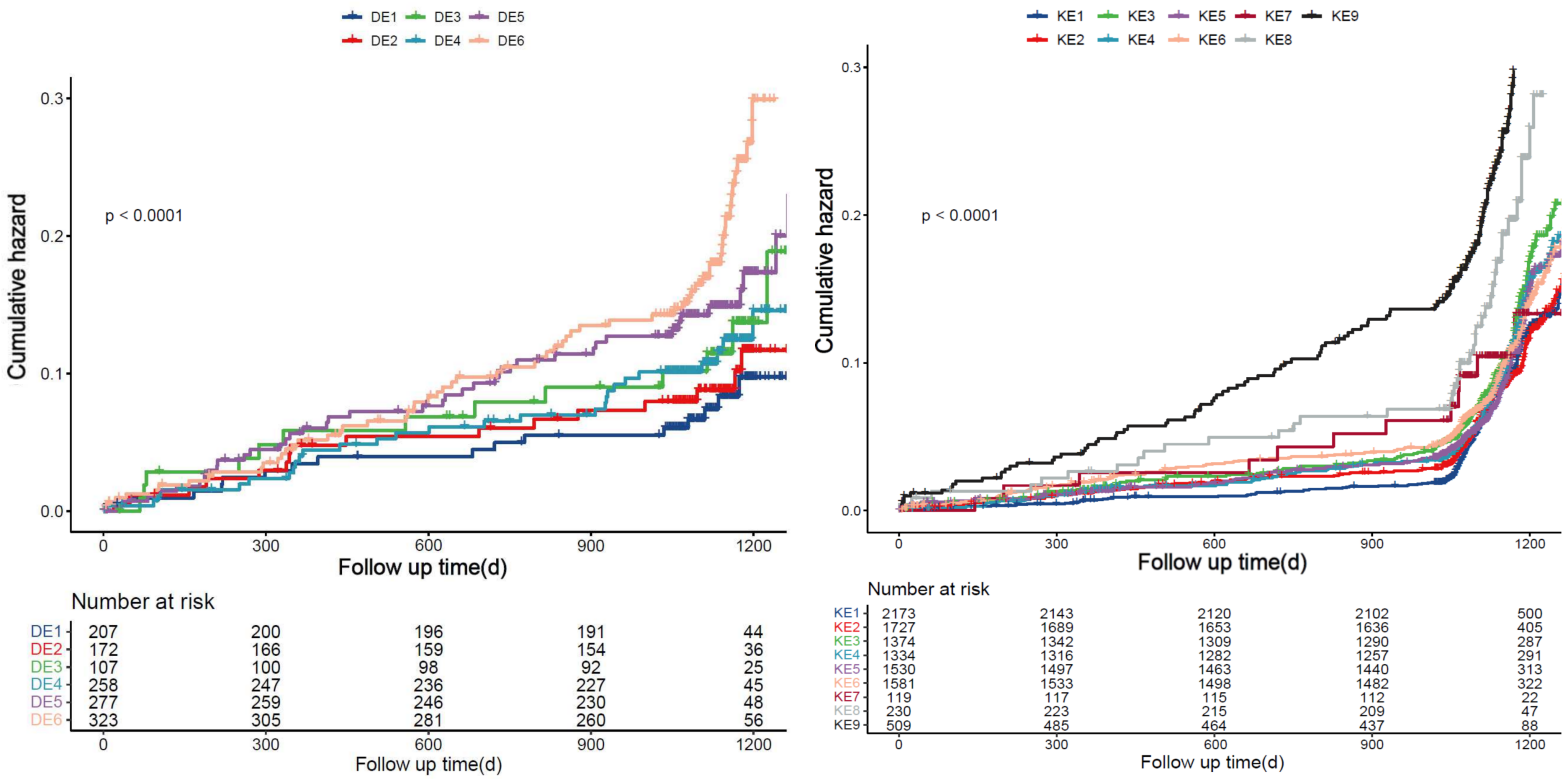
The Kaplan–Meier analysis for MACE in combined status of both DM and ET‐1 levels in CAD patients with impaired kidney function (A) and combined status of both kidney function and ET‐1 levels in CAD patients with DM. DE1, non‐DM plus low ET‐1 level; DE2, non‐DM plus medium ET‐1 level; DE3, non‐DM plus high ET‐1 level; DE4, DM plus low ET‐1 level; DE5, DM plus medium ET‐1 level; DE6, DM plus high ET‐1 level. KE1, eGFR ≥ 90 mL/min/1.73 m^2^ plus low ET‐1 level; KE2, eGFR ≥ 90 mL/min/1.73 m^2^ plus medium ET‐1 level; KE3, eGFR ≥ 90 mL/min/1.73 m^2^ plus high ET‐1 level; KE4, eGFR 60–90 mL/min/1.73 m^2^ plus low ET‐1 level; KE5, eGFR 0–90 mL/min/1.73 m^2^ plus medium ET‐1 level; KE6, eGFR 60–90 mL/min/1.73 m^2^ plus high ET‐1 level; KE7, eGFR ≤ 60 mL/min/1.73 m^2^ plus low ET‐1 level; KE8, eGFR ≤ 60 mL/min/1.73 m^2^ plus medium ET‐1 level; KE9, eGFR ≤ 60 mL/min/1.73 m^2^ plus high ET‐1 level. ET‐1, Endothelin‐1; MACE, major adverse cardiovascular events; DM, diabetes mellitus.

Crucially, when classifying patients with impaired kidney function according to their diabetes status and ET‐1 levels, individuals with diabetes and intermediate to high ET‐1 levels showed a markedly higher risk of MACE than the reference group, which consisted of those without diabetes and with low ET‐1 levels (Figure 3). Diabetes patients with medium ET‐1 levels and those with high ET‐1 levels were found to have a higher risk of MACE by multivariable regression analysis, with an adjusted HR of 2.082 (95% CI, 1.291–3.357, *p* < 0.001) and 2.134 (95% CI, 1.334–3.413, *p* < 0.001), respectively (Table 3). The risk of MACE was higher in non‐diabetic patients with high ET‐1 levels than in those with low ET‐1 levels; however, this link was not statistically significant when confounding variables were taken into account.

**TABLE 3:** Risk of MACE according to diabetic status and ET‐1 level from multivariate Cox regression analyses in CAD patients with impaired kidney function.

ET‐1	Events	Subjects	Robust model	Adjusted model
Non‐DM				
Low ET‐1	24	207	Reference	Reference
Medium ET‐1	21	172	1.016 (0.561–1.839)	0.903 (0.494–1.654)
High ET‐1	23	107	2.003 (1.130–3.552)^a^	1.618 (0.897–2.919)
DM				
Low ET‐1	37	258	1.345 (0.804–2.248)	1.353 (0.801–2.287)
Medium ET‐1	65	277	2.303 (1.441–3.682)^a^	2.082 (1.291–3.357)^a^
High ET‐1	99	323	2.976 (1.903–4.653)^a^	2.134 (1.334–3.413)^a^

*Note:* Adjusted Model adjusted for age, sex, body mass index (BMI), health status (acute coronary disease, smoking status, hypertension, dyslipidemia, family history of CAD), brain natriuretic peptide levels, medications at discharge (statins, aspirin, clopidogrel, ticagrelor, angiotensin II receptor blockers/angiotensin‐converting enzyme inhibitors, beta‐blockers, and calcium channel blockers), and angiographic characteristics (e.g., heavily calcified lesions, Syntax score, lesion length, chronic total occlusion, bifurcation, stent usage).

Abbreviations: DM, diabetes mellitus; ET‐1; Endothelin‐1; MACE, major adverse cardiovascular events.

^a^ *P* < 0.05.

Additionally, there was no discernible difference in the risk of cardiovascular death between the reference group and non‐diabetic patients with elevated ET‐1 levels. Similarly, non‐DM patients with high ET‐1 levels did not significantly differ in their risk of cardiovascular death from the reference group, but diabetic patients with medium and high ET‐1 levels had a statistically significant increased risk of cardiovascular mortality. Interestingly, the probability of a non‐fatal myocardial infarction did not differ statistically significantly from the reference group across all combined groups (Tables S5–S7 and Figure S2).

### 3.4 Kidney Function, ET‐1, and Clinical Outcomes

In patients with CAD and DM, MACE incidence rates differed by eGFR categories, recorded at 11.7%, 12.3%, and 23.4%, respectively (Figure 2). Among the groups, those with reduced kidney function had the lowest event‐free survival rates (log‐rank *p* < 0.001), according to Kaplan–Meier analysis. Furthermore, among the three groups, patients with the lowest event‐free survival had the highest ET‐1 levels (Figure S3).

Compared with patients with eGFR ≥ 90 mL/min/1.73 m^2^ and low ET‐1 levels, those with low eGFR and high ET‐1 levels had a considerably higher risk of MACE (*p* < 0.001) (Figure 3). Multivariate Cox regression analyses showed that patients with eGFR ≤ 60 mL/min/1.73 m^2^ and elevated ET‐1 levels had a 2.297‐fold (95% CI 1.822–2.895) increased risk of MACE, while those with medium ET‐1 levels had a 1.638‐fold (95% CI 1.179–2.275) increased risk. Patients with eGFR 60–90 mL/min/1.73 m^2^ and elevated ET‐1 levels had a 1.190‐fold (95% CI 1.025–1.333) increased MACE risk, whereas those with eGFR ≥ 90 mL/min/1.73 m^2^ and high ET‐1 levels had a 1.356‐fold (95% CI 1.117–1.646) increased risk (Table 4).

**TABLE 4:** Risk of MACE according to kidney function and ET‐1 status from multivariate Cox regression analyses in CAD patients with DM.

ET‐1	Events	Subjects	Robust model	Adjusted model
eGFR ≥ 90 mL/min/1.73 m^2^				
Low ET‐1	230	2173	Reference	Reference
Medium ET‐1	191	1727	1.085 (0.895–1.315)	1.048 (0.845–1.271)
High ET‐1	194	1374	1.453 (1.200–1.761)^a^	1.356 (1.117–1.646)^a^
60 < eGFR < 90 mL/min/1.73 m^2^				
Low ET‐1	161	1334	1.185 (0.968–1.449)	1.123 (0.914–1.379)
Medium ET‐1	179	1530	1.175 (0.966–1.429)	1.044 (0.853–1.277)
High ET‐1	207	1581	1.298 (1.075–1.567)^a^	1.190 (1.025–1.333)^a^
eGFR ≤ 60 mL/min/1.73 m^2^				
Low ET‐1	14	119	1.221 (0.712–2.095)	1.118 (0.645–1.936)
Medium ET‐1	46	230	2.015 (1.468–2.765)^a^	1.638 (1.179–2.275)^a^
High ET‐1	141	509	3.069 (2.487–3.787)^a^	2.297 (1.822–2.895)^a^

*Note:* Adjusted Model adjusted for age, sex, body mass index (BMI), health status (acute coronary disease, smoking status, hypertension, dyslipidemia, family history of CAD), brain natriuretic peptide levels, medications at discharge (statins, aspirin, clopidogrel, ticagrelor, angiotensin II receptor blockers/angiotensin‐converting enzyme inhibitors, beta‐blockers, and calcium channel blockers), and angiographic characteristics (e.g., heavily calcified lesions, Syntax score, lesion length, chronic total occlusion, bifurcation, stent usage).

Abbreviations: CV, cardiovascular; ET‐1; Endothelin‐1; MACE, major adverse cardiovascular events.

^a^ *P* < 0.05.

In terms of cardiovascular death and non‐fatal myocardial infarction, patients with eGFR ≤ 60 mL/min/1.73 m^2^ and intermediate to high levels of ET‐1 have a significantly higher cumulative hazard of cardiovascular death and non‐fatal myocardial infarction compared with other groups, showing statistical significance (*p* < 0.05) (Figure S4).

### 3.5 Non‐Linear Relationships

**FIGURE 4:**
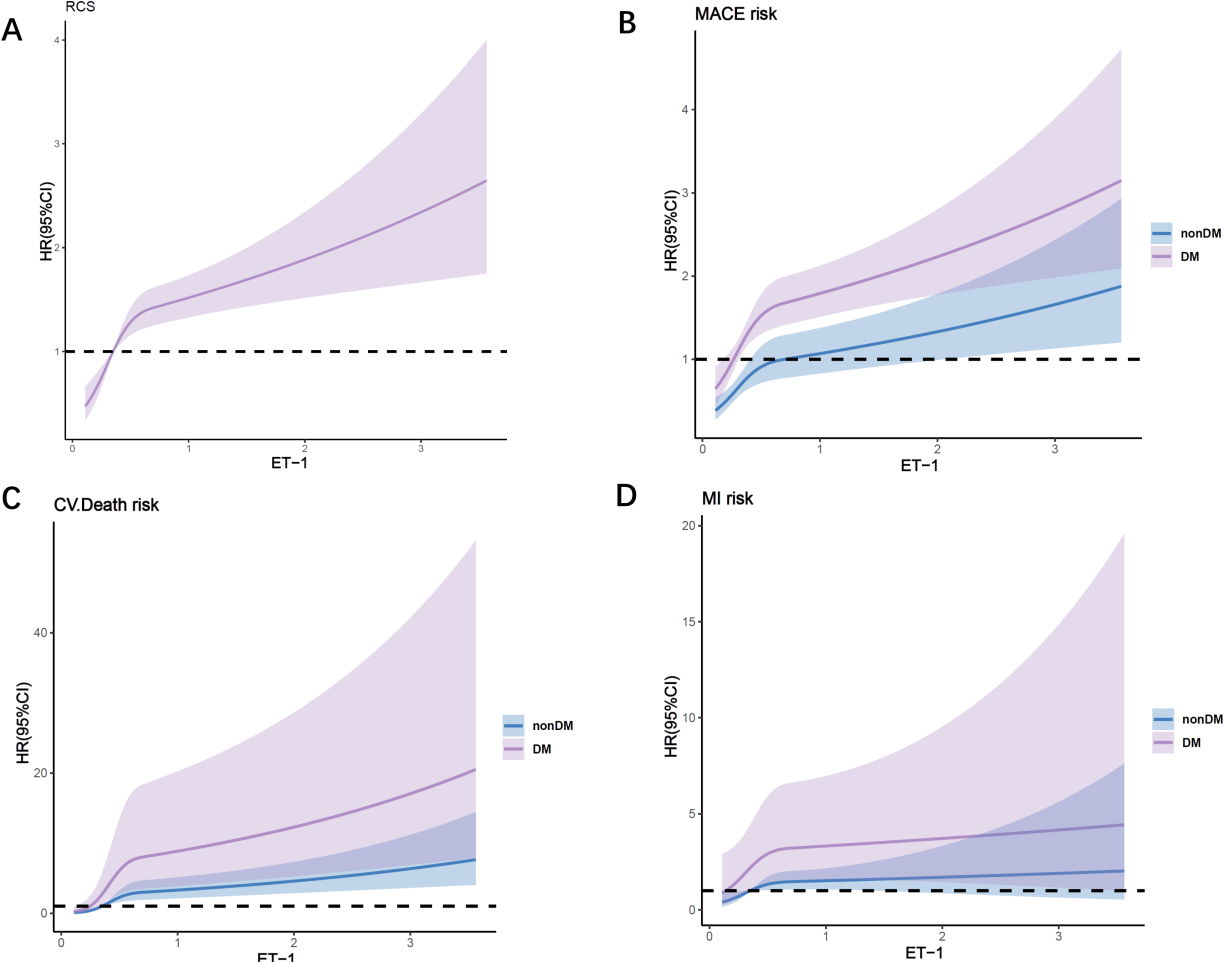
The restricted cubic spline analysis showed the relationships between ET‐1 and the risk of MACE in CAD patients with impaired kidney function. (A) MACE risk of CAD patients with impaired kidney function; (B) MACE risk of CAD patients with impaired kidney function in DM and non‐DM; (C) CV death risk of CAD patients with impaired kidney function in DM and non‐DM; (D) nonfatal MI risk of CAD patients with impaired kidney function in DM and non‐DM. ET‐1, Endothelin‐1; MACE, major adverse cardiovascular events; DM, diabetes mellitus; CV, cardiovascular; MI, myocardial infarction.

The Restrictive Cubic Splines (RCS) analysis demonstrated a non‐linear relationship between ET‐1 and MACE in both diabetic and non‐diabetic patients with CAD and impaired kidney function (P for nonlinear < 0.05) (Figure 4). Similarly, non‐linear associations were observed between ET‐1 and cardiovascular mortality and non‐fatal myocardial infarction. In patients with CAD and DM, there is a non‐linear relationship between the levels of ET‐1 and MACE (P for nonlinearity < 0.05). This conclusion remains consistent across patients with varying levels of eGFR (Figure S5).

### 3.6 Competing Risk Analyses

The competing risk analyses were used with the multivariable Fine‐Gray subdistribution hazard ratios (SHR) regarding non‐cardiac death as a competing risk. In CAD patients with impaired kidney function, a higher risk for MACE was found in the high ET‐1 level group (SHR 1.627, 95% CI 1.119–2.365, *p* = 0.011). Furthermore, diabetes patients with intermediate ET‐1 levels and those with high ET‐1 levels were found to have a higher risk of MACE with SHR of 1.961 (95% CI, 1.104–3.485, *p* = 0.022) and 2.192 (95% CI, 1.262–3.804, *p* = 0.005), respectively, which were consistent with Cox analysis.

In CAD patients with DM, ET‐1 was also positively associated with MACE (SHR 1.610, 95% CI 1.474–1.759, *p* < 0.001). CAD patients with impaired kidney functions with intermediate and high ET‐1 levels also had a higher MACE risk, with SHR of 3.509 (95% CI 1.841–6.689, *p* < 0.001) and 6.346 (95% CI 3.444–11.696, *p* < 0.001), respectively.

## 4 Discussion

This study examined the relationship between ET‐1 and MACE in CAD patients with differing renal function and with or without diabetes status undergoing PCI. Our study demonstrated that initial plasma ET‐1 levels independently predict MACE in CAD patients with impaired kidney function and CAD patients with DM, respectively. Furthermore, in CAD patients with impaired kidney function, elevated plasma ET‐1 levels were independently related to a higher risk of MACE, particularly in those with diabetes. In addition, elevated ET‐1 levels were independently associated with poorer cardiovascular outcomes in CAD patients with DM across differing renal function statuses. CAD patients who have elevated ET‐1 levels, renal insufficiency (eGFR ≤ 60 mL/min/1.73 m^2^) and DM faced the greatest risk for MACE. Our study underscored the predictive significance of ET‐1 for MACE in CAD patients who underwent PCI, especially in those with impaired kidney function and DM.

Because of its unique and long‐lasting effects, the 21‐amino acid peptide ET‐1 is recognized as the most potent endogenous vasoconstrictor [12, 13]. ET‐1 is the main endothelin isoform in the human cardiovascular system, primarily produced by the vascular endothelium and synthesized in organs such as the heart and kidneys. Continuous production of ET‐1 in the vasculature is essential for maintaining vascular tone [14] and blood pressure [15], thus playing a role in baseline vascular tension and the pathogenesis of several diseases such as hypertension [16, 17], CKD [18, 19], pulmonary arterial hypertension (PAH) [20, 21], and preeclampsia. ET‐1 exerts its effects via two G‐protein‐coupled receptors: the endothelin A (ETA) and endothelin B (ETB) receptors [22]. A common variation in a non‐coding gene sequence modulates the expression of EDN1 (the gene that encodes ET‐1) and has been associated with five prevalent vascular conditions: CAD, hypertension, migraine, carotid dissection, and fibromuscular dysplasia [23]. Therapeutic approaches target the endothelin system, leading to the development of several drugs. Selective ETA and dual ETA/ETB endothelin receptor antagonists (ERAs) [24] have been utilized in clinical settings to alleviate blood pressure and proteinuria in CKD patients and treat PAH. However, the available clinical evidence for their efficacy in heart failure and cardiovascular disease risk prevention remains unsatisfactory [25].

Although earlier studies have shown that ET‐1 is predictive of CAD patients [26, 27, 28], its role in secondary prevention remains less explored. Patients with CAD and glucose regulation abnormalities and impaired kidney function must undergo more meaningful prognostic biomarker evaluations [29]. Elevated levels of ET‐1, due to their proliferative, profibrotic, and pro‐inflammatory characteristics, may significantly impact cardiovascular complications [30]. Nonetheless, research regarding the value of ET‐1 in secondary prevention among patients with CAD and diabetes remains sparse as well as various kidney functions. Our research aims to investigate the therapeutic and prognostic value of this critical biomarker in patients with CAD, while further validating its role across populations with varying renal function and DM status. We found that in CAD patients with impaired kidney function and DM, the association between ET‐1 and MACE was significantly higher than in CAD patients with normal kidney function or without DM, indicating the important prognostic value of ET‐1 in these high‐risk patients. Endpoints such as nonfatal myocardial infarction, unplanned revascularization, and stroke involve competing risks with cardiovascular or non‐cardiovascular death, which may introduce bias into the results. After accounting for competing risks, the relationships between different outcomes did not significantly alter the main conclusions. The results of the competing risk analysis were consistent with those of the Cox regression analysis.

ET‐1 is essential in renal physiology and pathophysiology. It is not only involved in the regulation of glomerular hemodynamics but also induces cytoskeletal changes in podocytes, promoting further degeneration of the podocyte ultrastructure and leading to the degradation of the glomerular filtration barrier [22, 31]. This process exacerbates glomerular hypertension and accelerates the decline of functional renal mass. When renal dysfunction occurs, its metabolic pathway in the body is impaired, leading to different plasma levels compared with the general population. For example, similar to NT‐proBNP, patients with high‐risk CAD who concurrently have renal impairment or impaired glucose tolerance have different threshold values. Furthermore, ET‐1 is closely associated with coronary microvascular dysfunction [7, 32, 33]. The ET‐1 gene enhancer rs9349379‐G allele has been identified as a novel genetic risk locus for coronary microvascular impairment [34]. Elevated plasma levels of ET‐1 increase the risk of coronary microvascular dysfunction and the occurrence of no‐reflow phenomena after PCI [35]. DM is an effective inducer of microvascular dysfunction, characterized by reduced nitric oxide activity, increased production of reactive oxygen species, enhanced ET‐1 synthesis, impaired endothelial barrier function, and increased inflammatory activity and oxidative stress [36, 37, 38]. Our findings indicate that CAD patients with high levels of ET‐1 combined with impaired kidney function and DM have a significantly higher risk of adverse clinical events compared with the reference group. One possible explanation is that the synergistic effect of elevated ET‐1, DM, and impaired kidney function may contribute to the further deterioration of coronary microcirculation, which influences adverse cardiovascular outcomes.

Previous randomized, double‐blind, placebo‐controlled trials, named the SONAR study [39], suggest that a selective ERA may offer certain benefits to CKD patients. The DUET study confirmed that the dual antagonist sparsentan is more effective in reducing proteinuria compared with the isolated use of angiotensin receptor blockers, demonstrating good tolerability [40]. These trials indicate a close relationship between elevated ET‐1 levels and the deterioration of renal function, with relevant antagonists potentially providing positive effects for patients. This study investigates the combined impact of renal insufficiency and increased plasma ET‐1 levels on cardiovascular outcomes in patients with diabetes and CAD.

In this study, we further investigated the comprehensive risk assessment of ET‐1 and DM in impaired kidney function and CAD patients. In those patients with DM, elevated ET‐1 levels significantly increased the incidence of adverse cardiovascular clinical outcomes. Conversely, in CAD patients with impaired kidney function but without DM, ET‐1 did not have a statistically significant impact on MACE. However, this does not demonstrate that ET‐1 has no significant effect in non‐diabetic patients, as this may be attributable to the smaller number of non‐diabetic patients, particularly given the lower incidence of MACE in this group. Patients with CAD who have both renal impairment and high ET‐1 levels may benefit most from timely management and strategies to control blood glucose levels.

This study can assist clinicians in identifying high‐risk patients, facilitate resource allocation, and potentially provide clinical evidence to support further development of ET‐1 receptor antagonists or ET‐1 inhibitors. Several limitations must be acknowledged in our analysis. This study's generalizability and external validity are limited due to its single‐center design, with participants recruited from a major center in China. The study investigated the associations between ET‐1 levels and adverse outcomes in patients with CAD and varying levels of renal function, with or without diabetes. Overlap in patients with both renal impairment and diabetes is unavoidable; however, these analyses were conducted relatively independently, thus having minimal impact on the primary conclusions and overall analysis. Second, as an observational study, while we controlled for common potential confounders through multivariable adjustments, inherent biases due to unrecognized confounders might still influence the results. Since this was an observational study, it cannot establish causality. The investigation solely focused on baseline plasma ET‐1 levels, precluding a deeper exploration into how dynamic changes in ET‐1 concentrations might impact adverse outcomes. Additionally, this study focused exclusively on clinical aspects, and follow‐up time was relatively short. Future research should endeavor to elucidate the mechanisms that underlie the observed associations and evaluate the potential advantages of targeted therapies aimed at reducing ET‐1 levels.

## 5 Conclusion

Our research showed ET‐1 offered important prognostic information for CAD patients with impaired kidney function or with DM patients, with especially bad prognoses observed in those with elevated ET‐1 levels, renal dysfunction, and DM. This suggests that ET‐1 plays a pivotal role as a marker for high risk of poor prognosis in CAD patients with coexisting renal dysfunction and DM.

## Author Contributions

Z.Y., K.D., and W.Z. contributed to the study design. Z.Y. and E.X. contributed to data collection, manuscript writing, data processing, and figure mapping. Z.Y. and Y.D. contributed to the data proofreading. Z.Y. contributed to formal analysis; writing – original draft preparation. All authors have read and agreed to the published version of the manuscript.

## Consent

All participants provided written, informed consent.

## Conflicts of Interest

The authors declare no conflicts of interest.

## Supporting information


**Figure S1.** The Kaplan–Meier analysis for MACE risk in different ET‐1 groups (A) and DM status (B) in CAD patients with impaired kidney function. ET‐1, Endothelin‐1; DM, diabetes mellitus; MACE, major adverse cardiovascular events.
**Figure S2.** The Kaplan–Meier analysis for CV‐cause death (A), MI (B) in different combined status of both DM and ET‐1 levels. ET‐1, Endothelin‐1; DM, diabetes mellitus; CV, cardiovascular; MI, myocardial infarction.
**Figure S3.** The Kaplan–Meier analysis for MACE risk in different ET‐1 groups (A) and kidney function status (B) in CAD patients with DM. ET‐1, Endothelin‐1; DM, diabetes mellitus; MACE, major adverse cardiovascular events.
**Figure S4.** The Kaplan–Meier analysis for CV‐cause death(A), MI(B) in different combined status of both kidney function and ET‐1 levels. ET‐1, Endothelin‐1; DM, diabetes mellitus; CV, cardiovascular; MI, myocardial infarction.
**Figure S5.** The restricted cubic spline analysis showed the relationships between ET‐1 and the risk of MACE(A) and different kidney functions including eGFR ≥ 90 mL/min/1.73 m^2^ (B); eGFR 60–90 mL/min/1.73 m^2^ (C); eGFR ≤ 60 mL/min/1.73 m^2^ (D). MI(B) in the DM and non‐DM groups. ET‐1, Endothelin‐1; DM, diabetes mellitus; CV, cardiovascular; MI, myocardial infarction.

**Table S1.** Baseline characteristic of CAD patients with impaired kidney function in various ET‐1 level (full version).
**Table S2.** Baseline characteristic of CAD patients with impaired kidney function with or without DM.
**Table S3.** Baseline characteristics of CAD patients with diabetes in various kidney function stages.
**Table S4.** Univariate and multivariate Cox regression analyses for various adverse clinical outcomes risk related to ET‐1 levels in CAD patients with DM.
**Table S5.** Risk of all‐cause death according to diabetic status and ET‐1 level from multivariate Cox regression analyses in CAD patients with impaired kidney function.
**Table S6.** Risk of CV‐cause death according to diabetic status and ET‐1 level from multivariate Cox regression analyses in CAD patients with impaired kidney function.
**Table S7.** Risk of MI according to diabetic status and ET‐1 level from multivariate Cox regression analyses in CAD patients with impaired kidney function.

## Data Availability

The data that support the findings of this study are available on request from the corresponding author. The data are not publicly available due to privacy or ethical restrictions.
