# Supplementary figures and images for: The Role of Endothelin‐1, Kidney Function and Diabetes in Patients With Coronary Artery Disease Underwent Percutaneous Coronary Intervention
Source: J Diabetes. 2025 Jul 21;17(7):e70127. doi: 10.1111/1753-0407.70127 (PMC12280226; doi:10.1111/1753-0407.70127)

A

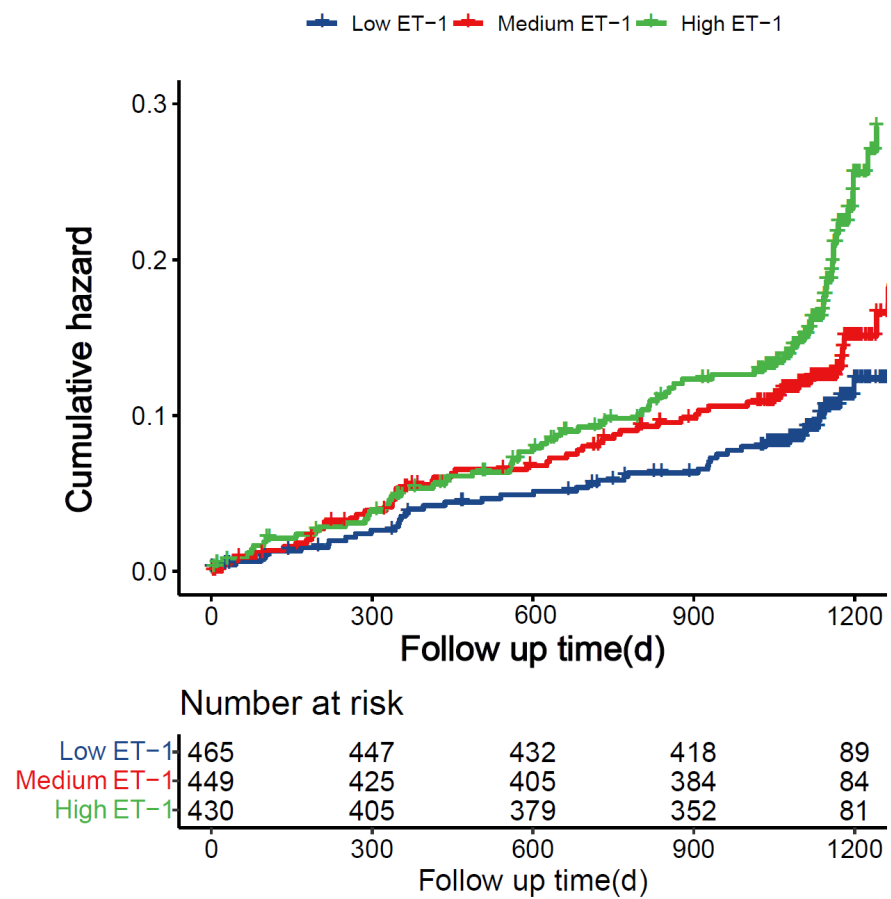

B

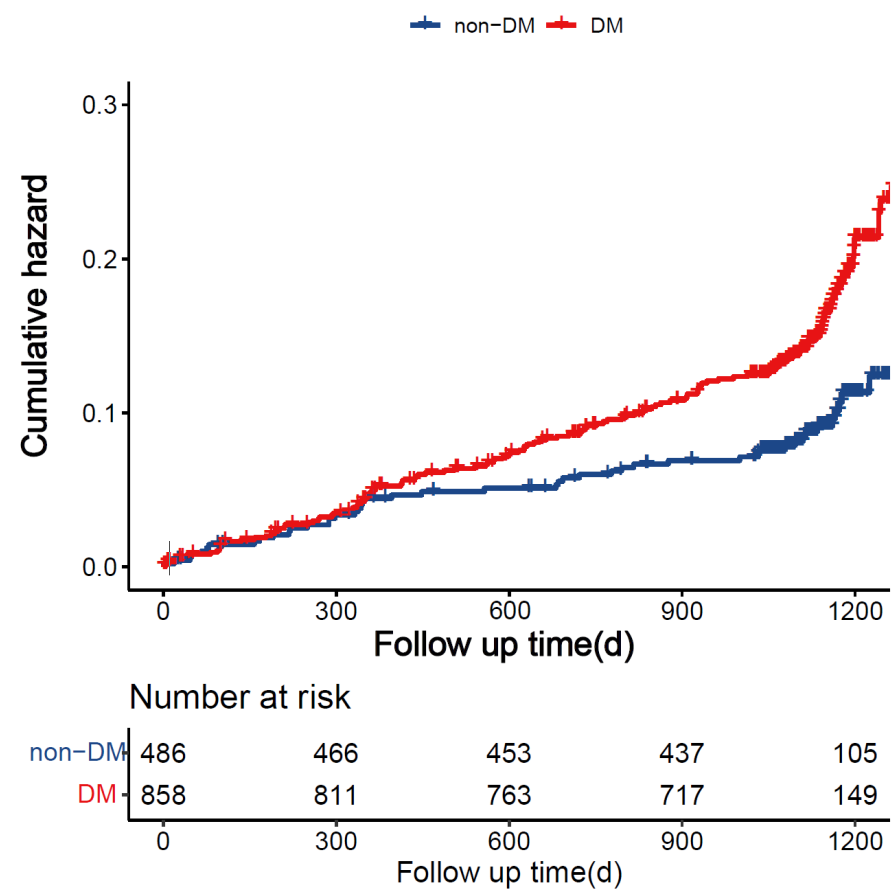

A

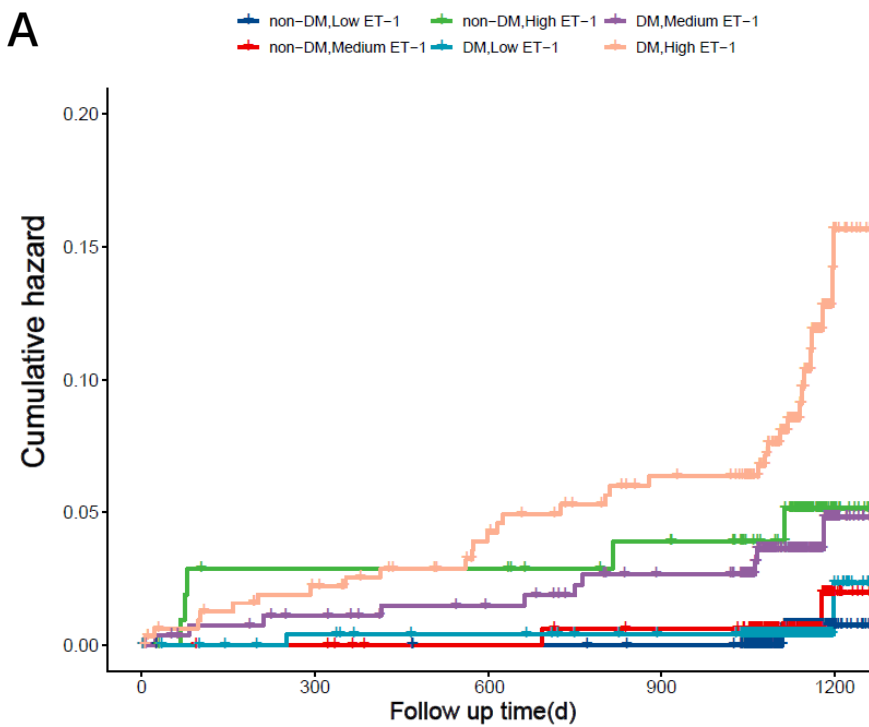

B

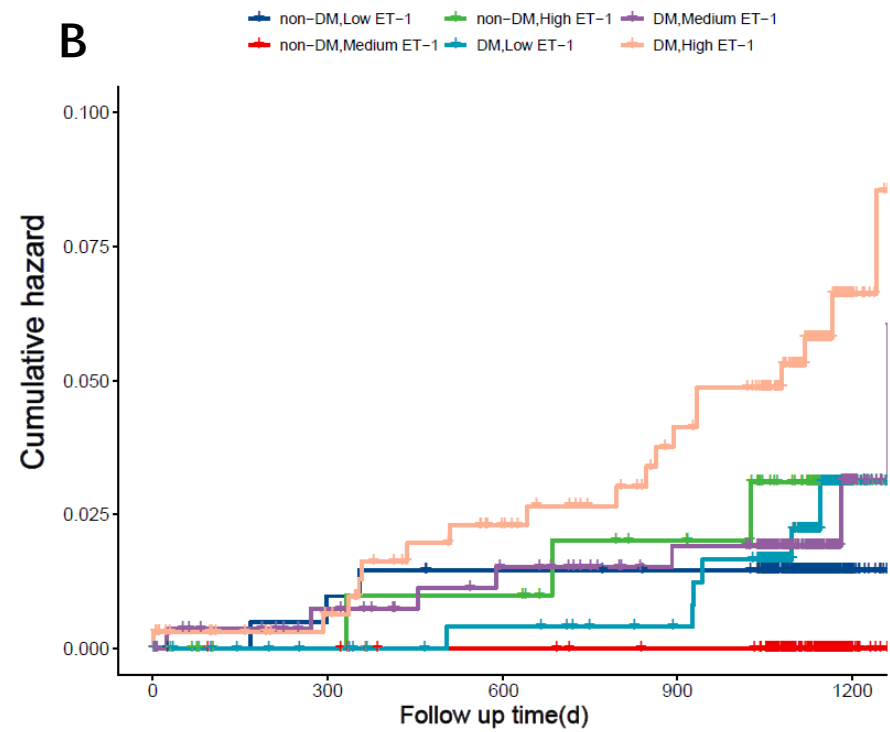

A

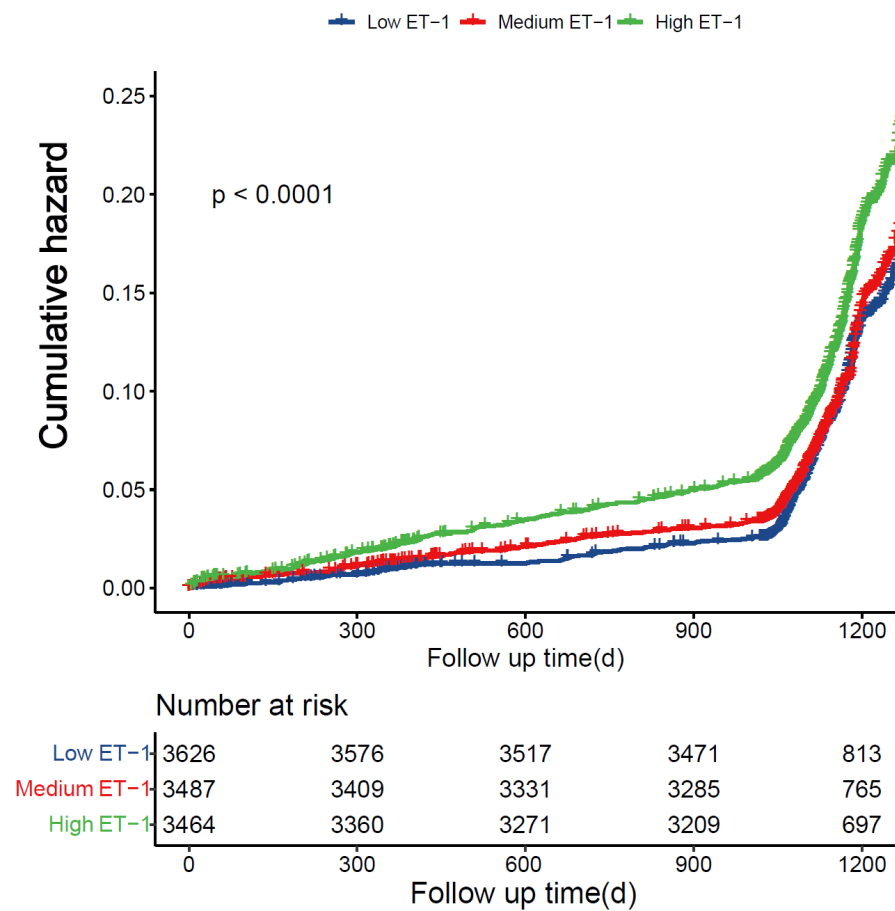

B

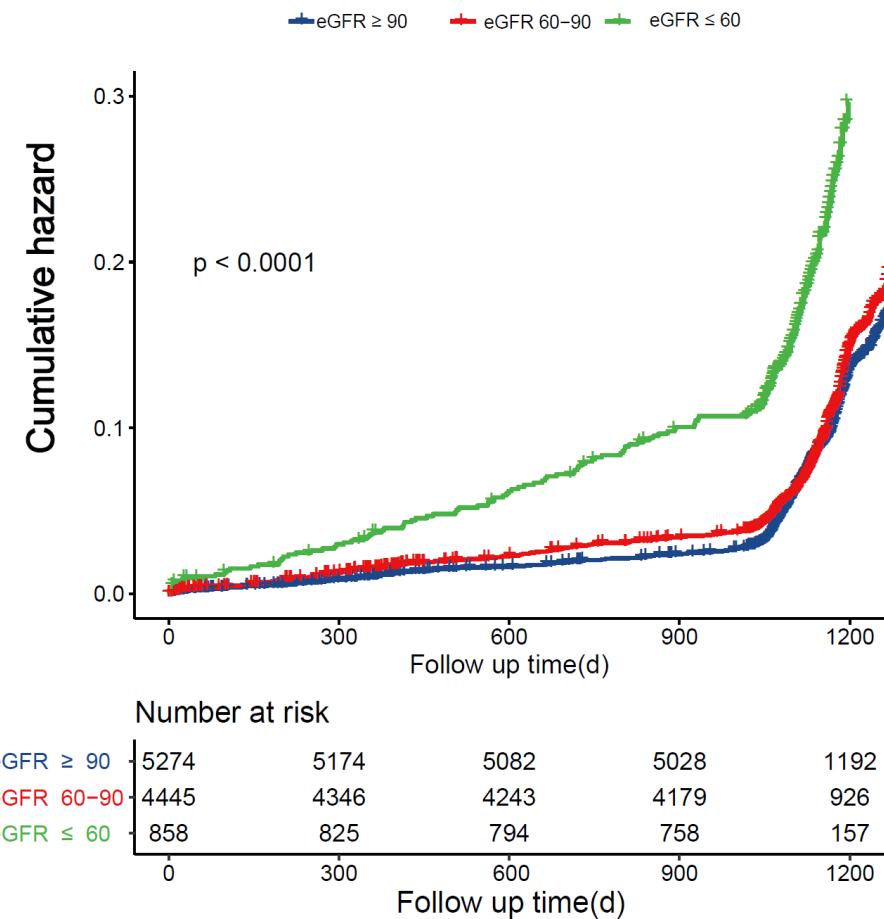

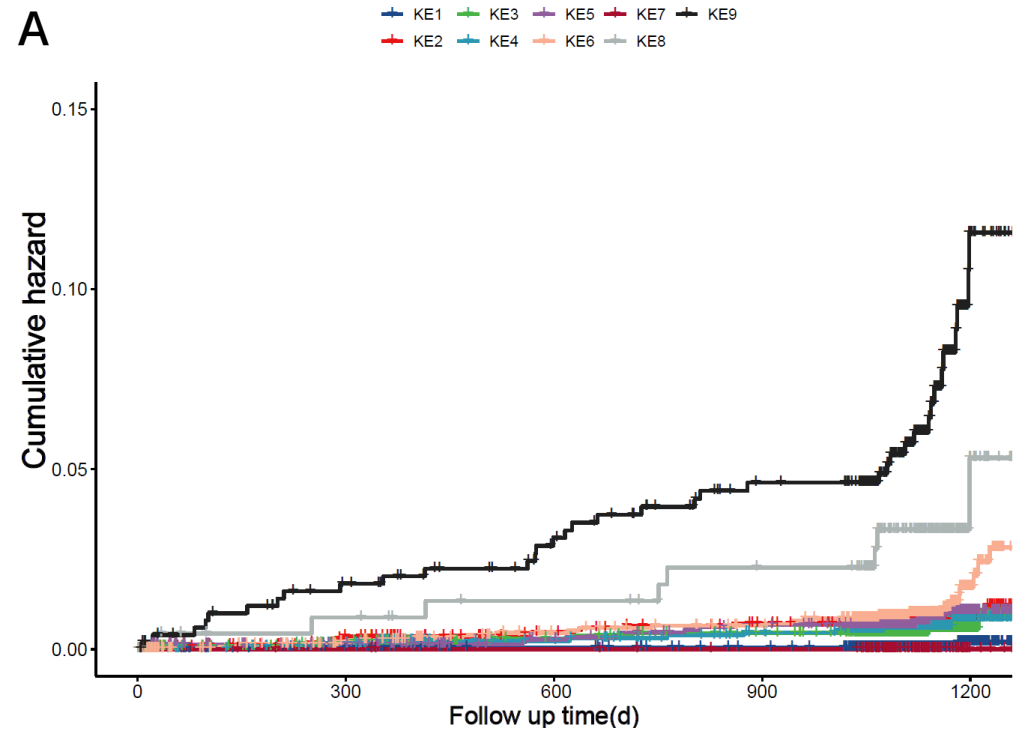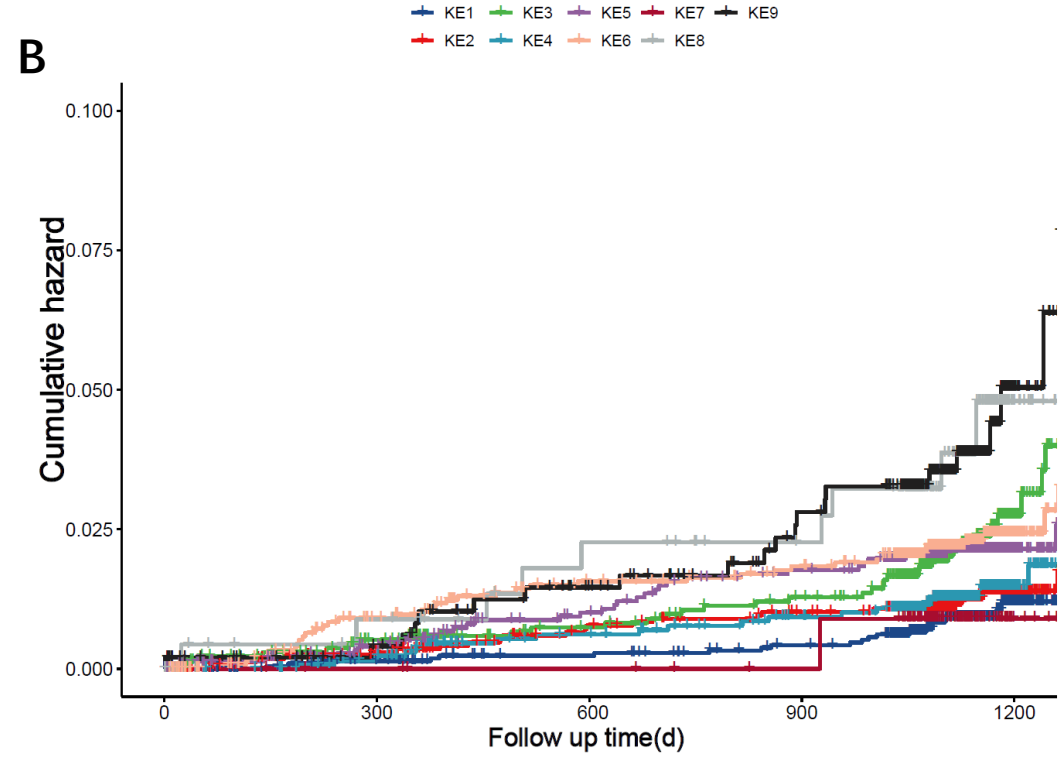

**A**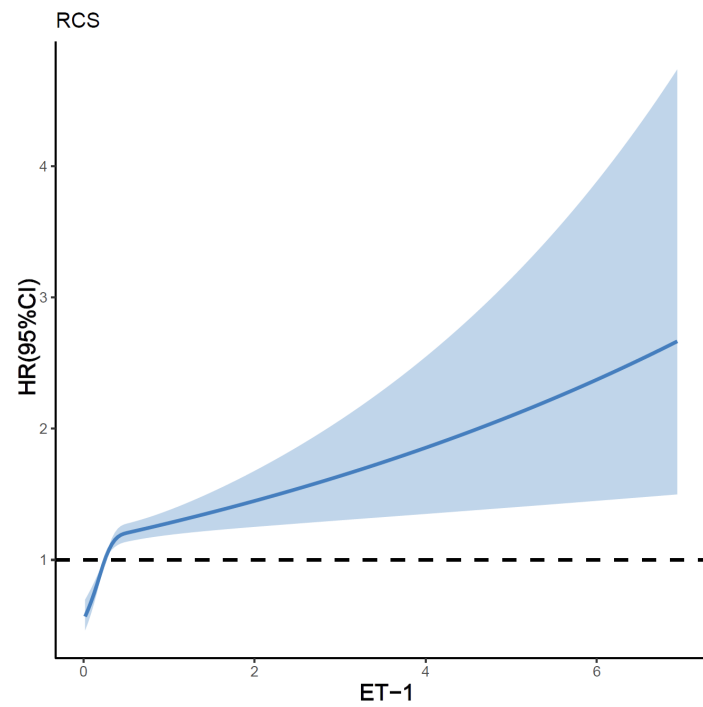**B**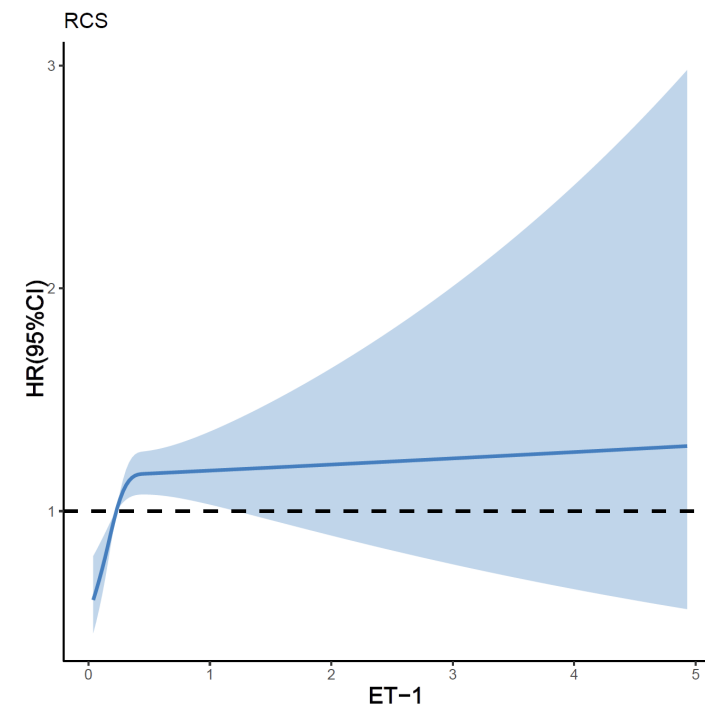**C**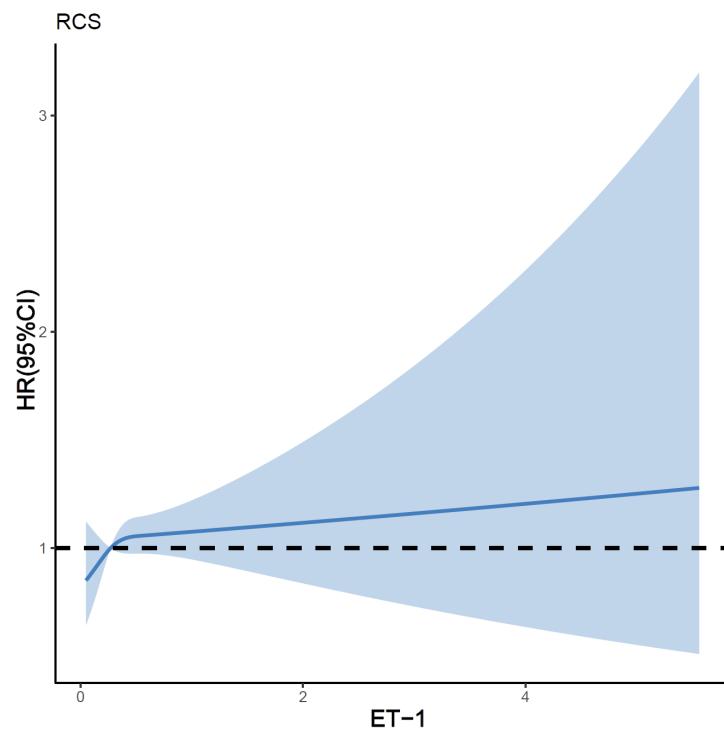**D**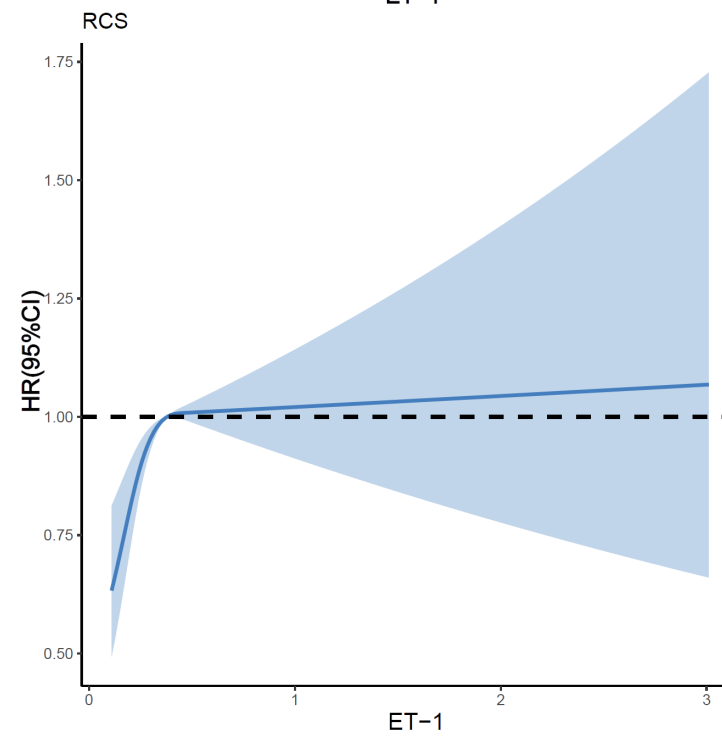

Supplement: Supplementary file 1 — Figure S1. The Kaplan–Meier analysis for MACE risk in different ET‐1 groups (A) and DM status (B) in CAD patients with impaired kidney function. ET‐1, Endothelin‐1; DM, diabetes mellitus; MACE, major adverse cardiovascular events. Figure S2. The Kaplan–Meier analysis for CV‐cause death (A), MI (B) in different combined status of both DM and ET‐1 levels. ET‐1, Endothelin‐1; DM, diabetes mellitus; CV, cardiovascular; MI, myocardial infarction. Figure S3. The Kaplan–Meier analysis for MACE risk in different ET‐1 groups (A) and kidney function status (B) in CAD patients with DM. ET‐1, Endothelin‐1; DM, diabetes mellitus; MACE, major adverse cardiovascular events. Figure S4. The Kaplan–Meier analysis for CV‐cause death(A), MI(B) in different combined status of both kidney function and ET‐1 levels. ET‐1, Endothelin‐1; DM, diabetes mellitus; CV, cardiovascular; MI, myocardial infarction. Figure S5. The restricted cubic spline analysis showed the relationships between ET‐1 and the risk of MACE(A) and different kidney functions including eGFR ≥ 90 mL/min/1.73 m2 (B); eGFR 60–90 mL/min/1.73 m2 (C); eGFR ≤ 60 mL/min/1.73 m2 (D). MI(B) in the DM and non‐DM groups. ET‐1, Endothelin‐1; DM, diabetes mellitus; CV, cardiovascular; MI, myocardial infarction. [file JDB-17-e70127-s002.pdf]
