# Supplementary material for: The Role of Endothelin‐1, Kidney Function and Diabetes in Patients With Coronary Artery Disease Underwent Percutaneous Coronary Intervention
Source: J Diabetes. 2025 Jul 21;17(7):e70127. doi: 10.1111/1753-0407.70127 (PMC12280226; doi:10.1111/1753-0407.70127)
Supplement: Supplementary file 2 — Table S1. Baseline characteristic of CAD patients with impaired kidney function in various ET‐1 level (full version). Table S2. Baseline characteristic of CAD patients with impaired kidney function with or without DM. Table S3. Baseline characteristics of CAD patients with diabetes in various kidney function stages. Table S4. Univariate and multivariate Cox regression analyses for various adverse clinical outcomes risk related to ET‐1 levels in CAD patients with DM. Table S5. Risk of all‐cause death according to diabetic status and ET‐1 level from multivariate Cox regression analyses in CAD patients with impaired kidney function. Table S6. Risk of CV‐cause death according to diabetic status and ET‐1 level from multivariate Cox regression analyses in CAD patients with impaired kidney function. Table S7. Risk of MI according to diabetic status and ET‐1 level from multivariate Cox regression analyses in CAD patients with impaired kidney function. [file JDB-17-e70127-s001.docx]

| **Supplementary Table 1 Baseline characteristic of CAD patients with impaired kidney function in various ET-1 level (full version).** | | | | | | |
| --- | --- | --- | --- | --- | --- | --- |
|  |  | Overall | Low ET-1 | Medium ET-1 | High ET-1 | P |
| n |  | 1344 | 465 | 449 | 430 |  |
| Male (%) |  | 799 (59.4) | 283 (60.9) | 277 (61.7) | 239 (55.6) | 0.136 |
| Age, y |  | 69.80 [63.20, 75.90] | 68.10 [61.90, 73.50] | 70.20 [63.40, 76.50] | 70.95 [64.62, 77.20] | <0.001 |
| BMI, kg/m2 |  | 25.69 [23.53, 27.70] | 25.71 [23.66, 27.68] | 25.65 [23.63, 27.72] | 25.60 [23.35, 27.88] | 0.88 |
| **Clinical presentation** | |  |  |  |  |  |
| ACS (%) |  | 924 (68.8) | 305 (65.6) | 296 (65.9) | 323 (75.1) | 0.003 |
| Atrial fibrillation (%) |  | 120 (8.9) | 21 (4.5) | 35 (7.8) | 64 (14.9) | <0.001 |
| prePCI (%) |  | 425 (31.6) | 135 (29.0) | 143 (31.8) | 147 (34.2) | 0.252 |
| preCABG (%) |  | 59 (4.4) | 13 (2.8) | 25 (5.6) | 21 (4.9) | 0.103 |
| DM (%) |  | 858 (63.8) | 258 (55.5) | 277 (61.7) | 323 (75.1) | <0.001 |
| Dyslipidemia (%) |  | 1049 (78.1) | 372 (80.0) | 353 (78.6) | 324 (75.3) | 0.229 |
| HTN (%) |  | 1105 (82.2) | 384 (82.6) | 374 (83.3) | 347 (80.7) | 0.583 |
| Family History CAD (%) |  | 137 (10.2) | 46 (9.9) | 50 (11.1) | 41 (9.5) | 0.71 |
| Smoking (%) |  | 705 (52.5) | 251 (54.0) | 235 (52.3) | 219 (50.9) | 0.658 |
| Anemia (%) |  | 91 (6.8) | 10 (2.2) | 31 (6.9) | 50 (11.6) | <0.001 |
| **Laboratory data** |  |  |  |  |  |  |
| LVEF,% |  | 61.00 [56.00, 65.00] | 62.00 [60.00, 65.00] | 61.50 [58.00, 65.00] | 60.00 [50.00, 63.00] | <0.001 |
| BNP, pg/ml |  | 326.30 [112.95, 1268.00] | 182.70 [76.50, 518.70] | 275.80 [117.80, 1020.00] | 966.20 [225.98, 3078.75] | <0.001 |
| ET, pmol/L |  | 0.35 [0.24, 0.50] | 0.22 [0.18, 0.24] | 0.35 [0.32, 0.40] | 0.60 [0.51, 0.81] | <0.001 |
| Glu, mmol/L |  | 6.59 [5.45, 8.75] | 6.03 [5.34, 7.91] | 6.55 [5.45, 8.50] | 7.36 [5.72, 10.12] | <0.001 |
| HbA1C, % |  | 6.50 [5.90, 7.60] | 6.30 [5.80, 7.30] | 6.50 [5.90, 7.50] | 6.70 [6.00, 7.88] | <0.001 |
| TC, mmol/L |  | 4.00 [3.37, 4.77] | 4.01 [3.41, 4.71] | 4.11 [3.41, 4.84] | 3.86 [3.28, 4.67] | 0.05 |
| TG, mmol/L |  | 1.64 [1.21, 2.27] | 1.58 [1.17, 2.16] | 1.72 [1.28, 2.40] | 1.63 [1.20, 2.22] | 0.094 |
| LDL, mmol/L |  | 2.33 [1.82, 3.00] | 2.27 [1.82, 2.93] | 2.43 [1.89, 3.06] | 2.29 [1.77, 2.99] | 0.113 |
| HDL, mmol/L |  | 1.07 [0.89, 1.27] | 1.12 [0.94, 1.32] | 1.08 [0.90, 1.26] | 1.00 [0.84, 1.22] | <0.001 |
| UA, mmol/L |  | 411.26 [349.32, 486.66] | 409.82 [347.90, 481.65] | 406.00 [344.91, 470.64] | 419.97 [358.82, 508.79] | 0.024 |
| Ka, mmol/L |  | 4.29 [4.01, 4.57] | 4.32 [4.04, 4.56] | 4.30 [4.02, 4.59] | 4.26 [3.93, 4.57] | 0.238 |
| Na, mmol/L |  | 139.51 [137.70, 141.30] | 139.90 [138.10, 141.40] | 139.74 [138.11, 141.30] | 138.81 [136.69, 141.00] | <0.001 |
| CRP, mmol/L |  | 3.79 [2.35, 7.43] | 3.29 [2.21, 5.80] | 3.47 [2.25, 6.72] | 5.41 [2.80, 13.35] | <0.001 |
| HBG, g/dL |  | 4.45 [4.02, 4.92] | 4.65 [4.25, 5.07] | 4.47 [4.10, 4.91] | 4.20 [3.79, 4.67] | <0.001 |
| PLT, K/uL |  | 222.00 [183.00, 266.00] | 219.00 [185.00, 266.00] | 228.00 [192.00, 267.00] | 217.00 [174.00, 266.00] | 0.011 |
| WBC, K/uL |  | 7.05 [5.89, 8.38] | 6.79 [5.89, 7.92] | 7.02 [5.86, 8.20] | 7.42 [5.92, 9.01] | <0.001 |
| ALT, mmol/L |  | 21.00 [14.00, 31.00] | 22.00 [16.00, 32.00] | 20.00 [14.00, 29.00] | 20.00 [14.00, 31.00] | 0.059 |
| AST, mmol/L |  | 22.00 [18.00, 29.00] | 22.00 [18.00, 27.00] | 22.00 [18.00, 28.00] | 23.00 [18.00, 37.75] | 0.04 |
| BUN, mmol/L |  | 7.70 [6.30, 9.30] | 7.40 [6.16, 8.97] | 7.60 [6.30, 9.10] | 8.16 [6.55, 10.04] | <0.001 |
| CR |  | 1.42 (0.45) | 1.35 (0.27) | 1.40 (0.29) | 1.51 (0.67) | <0.001 |
| eGFR, mL/min/1.73m2 |  | 50.32 (8.80) | 52.64 (6.55) | 50.53 (7.96) | 47.59 (10.79) | <0.001 |
| **Angiographic and PCI data** | | |  |  |  |  |
| Heavily calcified (%) |  | 77 (5.7) | 27 (5.8) | 28 (6.2) | 22 (5.1) | 0.772 |
| LM (%) |  | 63 (4.7) | 18 (3.9) | 24 (5.3) | 21 (4.9) | 0.558 |
| LAD (%) |  | 538 (40.0) | 187 (40.2) | 182 (40.5) | 169 (39.3) | 0.928 |
| LCX (%) |  | 202 (15.0) | 87 (18.7) | 54 (12.0) | 61 (14.2) | 0.015 |
| RCA (%) |  | 517 (38.5) | 171 (36.8) | 184 (41.0) | 162 (37.7) | 0.392 |
| Syntax |  | 14.00 [7.00, 21.00] | 13.00 [7.00, 20.00] | 13.00 [7.00, 20.00] | 14.75 [8.00, 22.00] | 0.162 |
| Triple vessel (%) |  | 750 (55.8) | 241 (51.8) | 264 (58.8) | 245 (57.0) | 0.088 |
| MLD, mm |  | 0.30 [0.04, 0.50] | 0.30 [0.15, 0.52] | 0.30 [0.14, 0.50] | 0.25 [0.00, 0.46] | <0.001 |
| DS, mm |  | 90.00 [80.00, 99.00] | 90.00 [80.00, 95.00] | 90.00 [80.00, 95.00] | 90.00 [80.00, 100.00] | <0.001 |
| TIMI (%) | 0 | 272 (20.2) | 73 (15.7) | 82 (18.3) | 117 (27.2) | <0.001 |
|  | 1 | 55 (4.1) | 22 (4.7) | 9 (2.0) | 24 (5.6) |  |
|  | 2 | 147 (10.9) | 65 (14.0) | 41 (9.1) | 41 (9.5) |  |
|  | 3 | 870 (64.7) | 305 (65.6) | 317 (70.6) | 248 (57.7) |  |
| B2_C_lesion (%) |  | 996 (74.1) | 334 (71.8) | 336 (74.8) | 326 (75.8) | 0.362 |
| Diffused Range (%) |  | 777 (57.8) | 272 (58.5) | 259 (57.7) | 246 (57.2) | 0.925 |
| Extremely Tortuosity (%) |  | 48 (3.6) | 20 (4.3) | 19 (4.2) | 9 (2.1) | 0.134 |
| Angulated (%) |  | 445 (33.1) | 159 (34.2) | 160 (35.6) | 126 (29.3) | 0.113 |
| Irregular (%) |  | 1045 (77.8) | 343 (73.8) | 359 (80.0) | 343 (79.8) | 0.038 |
| CTO (%) |  | 138 (10.3) | 43 (9.2) | 48 (10.7) | 47 (10.9) | 0.665 |
| Ostial (%) |  | 189 (14.1) | 59 (12.7) | 80 (17.8) | 50 (11.6) | 0.018 |
| Bifurcation (%) |  | 242 (18.0) | 82 (17.6) | 87 (19.4) | 73 (17.0) | 0.63 |
| Stent length,mm |  | 28.00 [18.00, 41.00] | 28.00 [18.00, 39.00] | 30.00 [18.00, 44.00] | 26.00 [18.00, 40.00] | 0.243 |
| Stent num |  | 1.00 [1.00, 2.00] | 1.00 [1.00, 2.00] | 2.00 [1.00, 2.00] | 1.00 [1.00, 2.00] | 0.581 |
| Stent (%) |  | 1196 (89.0) | 408 (87.7) | 398 (88.6) | 390 (90.7) | 0.354 |
| **Medications at discharge** | | |  |  |  |  |
| ARB/ACEI (%) |  | 424 (31.5) | 173 (37.2) | 142 (31.6) | 109 (25.3) | 0.001 |
| CCB (%) |  | 703 (52.3) | 237 (51.0) | 260 (57.9) | 206 (47.9) | 0.009 |
| NIT (%) |  | 1291 (96.1) | 451 (97.0) | 439 (97.8) | 401 (93.3) | 0.001 |
| beta (%) |  | 1205 (89.7) | 411 (88.4) | 402 (89.5) | 392 (91.2) | 0.393 |
| statin (%) |  | 1327 (98.7) | 463 (99.6) | 444 (98.9) | 420 (97.7) | 0.038 |
| Aspirin (%) |  | 1331 (99.0) | 461 (99.1) | 443 (98.7) | 427 (99.3) | 0.6 |
| Clopidogrel (%) |  | 1327 (98.7) | 463 (99.6) | 442 (98.4) | 422 (98.1) | 0.127 |
| Ticagrelor (%) |  | 217 (16.1) | 70 (15.1) | 63 (14.0) | 84 (19.5) | 0.063 |
| LMWH (%) |  | 858 (63.8) | 296 (63.7) | 258 (57.5) | 304 (70.7) | <0.001 |
| Oral hypoglycaemics (%) |  | 389 (28.9) | 133 (28.6) | 118 (26.3) | 138 (32.1) | 0.161 |
| Insulin (%) |  | 215 (16.0) | 64 (13.8) | 71 (15.8) | 80 (18.6) | 0.141 |
| DM Dietary Control (%) |  | 44 (3.3) | 14 (3.0) | 18 (4.0) | 12 (2.8) | 0.553 |
|  |  |  |  |  |  |  |
| ET-1; Endothelin-1; BMI, body mass index; WBC, white blood cell; HBG, hemoglobin; PLT, platelet; eGFR, estimated glomerular filtration rate; ACS, acute coronary syndrome; DM, diabetes mellitus；PCI, percutaneous coronary intervention; TG, triglycerides; TC, total cholesterol; LDL-C, low-density lipoprotein cholesterol; HDL-C, high-density lipoprotein cholesterol；MLD, minimal lumen diameter; TIMI, Thrombolysis In Myocardial Infarction; LM, left main coronary artery; SYNTAX, synergy between percutaneous coronary intervention with TAXUS and cardiac surgery; BNP, brain natriuretic peptide; CCB, calcium channel blockers; ARB/ACEI, angiotensin II receptor blockers/angiotensin converting enzyme inhibitors; LMWH, low molecular weight heparin; CTO, chronic total occlusion. | | | | | | |

| **Supplementary Table2 Baseline characteristic of CAD patients with impaired kidney function with or without DM.** | | | | | |
| --- | --- | --- | --- | --- | --- |
|  |  | Overall | non-DM | DM | p |
| n |  | 1344 | 486 | 858 |  |
| Male (%) |  | 799 (59.4) | 323 (66.5) | 476 (55.5) | <0.001 |
| Age, y |  | 69.80 [63.20, 75.90] | 70.20 [62.62, 76.70] | 69.60 [63.60, 75.47] | 0.656 |
| BMI, kg/m2 |  | 25.69 [23.53, 27.70] | 25.25 [23.20, 27.21] | 25.95 [23.85, 27.88] | <0.001 |
| **Clinical presentation** |  |  |  |  |  |
| ACS (%) |  | 924 (68.8) | 325 (66.9) | 599 (69.8) | 0.291 |
| Atrial fibrillation (%) |  | 120 (8.9) | 40 (8.2) | 80 (9.3) | 0.565 |
| prePCI (%) |  | 425 (31.6) | 127 (26.1) | 298 (34.7) | 0.001 |
| preCABG (%) |  | 59 (4.4) | 12 (2.5) | 47 (5.5) | 0.014 |
| Dyslipidemia (%) |  | 1049 (78.1) | 363 (74.7) | 686 (80.0) | 0.03 |
| HTN (%) |  | 1105 (82.2) | 391 (80.5) | 714 (83.2) | 0.23 |
| Family History CAD (%) |  | 137 (10.2) | 57 (11.7) | 80 (9.3) | 0.192 |
| Smoking (%) |  | 705 (52.5) | 280 (57.6) | 425 (49.5) | 0.005 |
| Anemia (%) |  | 91 (6.8) | 28 (5.8) | 63 (7.3) | 0.319 |
| **Laboratory data** |  |  |  |  |  |
| LVEF,% |  | 61.00 [56.00, 65.00] | 62.00 [58.00, 65.00] | 60.00 [55.00, 65.00] | 0.008 |
| BNP, pg/ml |  | 326.30 [112.95, 1268.00] | 253.55 [89.83, 896.55] | 388.85 [122.55, 1477.00] | <0.001 |
| ET, pmol/L |  | 0.35 [0.24, 0.50] | 0.31 [0.22, 0.42] | 0.38 [0.25, 0.53] | <0.001 |
| Glu, mmol/L |  | 6.59 [5.45, 8.75] | 5.39 [4.97, 5.81] | 8.06 [6.60, 10.23] | <0.001 |
| HbA1C,% |  | 6.50 [5.90, 7.60] | 5.90 [5.60, 6.10] | 7.20 [6.50, 8.17] | <0.001 |
| TC, mmol/L |  | 4.00 [3.37, 4.77] | 3.99 [3.39, 4.72] | 4.00 [3.35, 4.80] | 0.893 |
| TG, mmol/L |  | 1.64 [1.21, 2.27] | 1.54 [1.14, 2.16] | 1.70 [1.27, 2.33] | 0.001 |
| LDL, mmol/L |  | 2.33 [1.82, 3.00] | 2.32 [1.85, 2.97] | 2.33 [1.81, 3.00] | 0.94 |
| HDL, mmol/L |  | 1.07 [0.89, 1.27] | 1.10 [0.94, 1.29] | 1.05 [0.87, 1.26] | 0.001 |
| UA, mmol/L |  | 411.26 [349.32, 486.66] | 426.60 [366.31, 505.19] | 399.71 [340.36, 473.80] | <0.001 |
| Ka, mmol/L |  | 4.29 [4.01, 4.57] | 4.26 [3.99, 4.54] | 4.31 [4.01, 4.60] | 0.162 |
| Na, mmol/L |  | 139.51 [137.70, 141.30] | 139.90 [138.20, 141.70] | 139.30 [137.48, 141.00] | <0.001 |
| CRP, mmol/L |  | 3.79 [2.35, 7.43] | 3.65 [2.32, 6.72] | 3.87 [2.39, 8.09] | 0.176 |
| HBG, g/dL |  | 4.45 [4.02, 4.92] | 4.53 [4.04, 4.99] | 4.42 [4.01, 4.85] | 0.009 |
| PLT, K/uL |  | 222.00 [183.00, 266.00] | 218.50 [180.00, 262.00] | 224.00 [185.00, 267.75] | 0.097 |
| WBC, K/uL |  | 7.05 [5.89, 8.38] | 6.54 [5.66, 7.87] | 7.26 [6.04, 8.65] | <0.001 |
| ALT, mmol/L |  | 21.00 [14.00, 31.00] | 21.00 [14.00, 30.00] | 21.00 [14.00, 31.00] | 0.837 |
| AST, mmol/L |  | 22.00 [18.00, 29.00] | 23.00 [19.00, 29.00] | 22.00 [18.00, 29.00] | 0.143 |
| BUN, mmol/L |  | 7.70 [6.30, 9.30] | 7.26 [6.04, 9.00] | 7.92 [6.51, 9.43] | <0.001 |
| CR |  | 1.42 (0.45) | 1.42 (0.39) | 1.42 (0.48) | 0.902 |
| eGFR, mL/min/1.73m2 |  | 50.32 (8.80) | 50.90 (8.17) | 49.99 (9.13) | 0.071 |
| **Angiographic and PCI data** | |  |  |  |  |
| Heavily calcified (%) |  | 77 (5.7) | 27 (5.6) | 50 (5.8) | 0.933 |
| LM (%) |  | 63 (4.7) | 24 (4.9) | 39 (4.5) | 0.847 |
| LAD (%) |  | 538 (40.0) | 204 (42.0) | 334 (38.9) | 0.299 |
| LCX (%) |  | 202 (15.0) | 74 (15.2) | 128 (14.9) | 0.942 |
| RCA (%) |  | 517 (38.5) | 174 (35.8) | 343 (40.0) | 0.146 |
| Syntax |  | 14.00 [7.00, 21.00] | 12.50 [6.00, 20.00] | 14.50 [8.00, 21.88] | 0.003 |
| Triple vessel (%) |  | 750 (55.8) | 243 (50.0) | 507 (59.1) | 0.002 |
| MLD, mm |  | 0.30 [0.04, 0.50] | 0.30 [0.13, 0.50] | 0.28 [0.03, 0.50] | 0.229 |
| DS, mm |  | 90.00 [80.00, 99.00] | 90.00 [80.00, 95.00] | 90.00 [80.00, 99.00] | 0.567 |
| TIMI (%) | 0 | 272 (20.2) | 82 (16.9) | 190 (22.1) | 0.034 |
|  | 1 | 55 (4.1) | 27 (5.6) | 28 (3.3) |  |
|  | 2 | 147 (10.9) | 53 (10.9) | 94 (11.0) |  |
|  | 3 | 870 (64.7) | 324 (66.7) | 546 (63.6) |  |
| B2_C_lesion (%) |  | 996 (74.1) | 356 (73.3) | 640 (74.6) | 0.635 |
| Diffused Range (%) |  | 777 (57.8) | 274 (56.4) | 503 (58.6) | 0.457 |
| Extremely Tortuosity (%) |  | 48 (3.6) | 23 (4.7) | 25 (2.9) | 0.116 |
| Angulated (%) |  | 445 (33.1) | 185 (38.1) | 260 (30.3) | 0.004 |
| Irregular (%) |  | 1045 (77.8) | 371 (76.3) | 674 (78.6) | 0.384 |
| CTO (%) |  | 138 (10.3) | 41 (8.4) | 97 (11.3) | 0.116 |
| Ostial (%) |  | 189 (14.1) | 65 (13.4) | 124 (14.5) | 0.642 |
| Bifurcation (%) |  | 242 (18.0) | 95 (19.5) | 147 (17.1) | 0.302 |
| Stent length, mm |  | 28.00 [18.00, 41.00] | 29.00 [18.00, 40.00] | 28.00 [18.00, 41.75] | 0.473 |
| Stent number |  | 1.00 [1.00, 2.00] | 1.00 [1.00, 2.00] | 1.00 [1.00, 2.00] | 0.832 |
| Stent (%) |  | 1196 (89.0) | 437 (89.9) | 759 (88.5) | 0.466 |
| **Medications at discharge** |  |  |  |  |  |
| ARB/ACEI (%) |  | 424 (31.5) | 141 (29.0) | 283 (33.0) | 0.149 |
| CCB (%) |  | 703 (52.3) | 236 (48.6) | 467 (54.4) | 0.044 |
| NIT (%) |  | 1291 (96.1) | 473 (97.3) | 818 (95.3) | 0.098 |
| beta (%) |  | 1205 (89.7) | 427 (87.9) | 778 (90.7) | 0.125 |
| statin (%) |  | 1327 (98.7) | 480 (98.8) | 847 (98.7) | 1 |
| Aspirin (%) |  | 1331 (99.0) | 481 (99.0) | 850 (99.1) | 1 |
| Clopidogrel (%) |  | 1327 (98.7) | 480 (98.8) | 847 (98.7) | 1 |
| Ticagrelor (%) |  | 217 (16.1) | 77 (15.8) | 140 (16.3) | 0.881 |
| LMWH (%) |  | 858 (63.8) | 296 (60.9) | 562 (65.5) | 0.104 |
| Oral hypoglycaemics (%) |  | 389 (28.9) | 1 (0.2) | 388 (45.2) | <0.001 |
| Insulin (%) |  | 215 (16.0) | 1 (0.2) | 214 (24.9) | <0.001 |
| DM Dietary Control (%) |  | 44 (3.3) | 3 (0.6) | 41 (4.8) | <0.001 |
|  |  |  |  |  |  |
| ET-1; Endothelin-1; BMI, body mass index; WBC, white blood cell; HBG, hemoglobin; PLT, platelet; eGFR, estimated glomerular filtration rate; ACS, acute coronary syndrome; DM, diabetes mellitus；PCI, percutaneous coronary intervention; TG, triglycerides; TC, total cholesterol; LDL-C, low-density lipoprotein cholesterol; HDL-C, high-density lipoprotein cholesterol; MLD, minimal lumen diameter; TIMI, Thrombolysis In Myocardial Infarction; LM, left main coronary artery; SYNTAX, synergy between percutaneous coronary intervention with TAXUS and cardiac surgery; BNP, brain natriuretic peptide; CCB, calcium channel blockers; ARB/ACEI, angiotensin II receptor blockers/angiotensin converting enzyme inhibitors; LMWH, low molecular weight heparin; CTO, chronic total occlusion. | | | | | |

| **Supplementary Table 3 Baseline characteristics of CAD patients** **with diabetes in various kidney function stages.** | | | | | | |
| --- | --- | --- | --- | --- | --- | --- |
|  |  | Overall | eGFR≥90 mL/min/1.73 m² | 60<eGFR<90 mL/min/1.73 m² | eGFR≤60 mL/min/1.73 m² | P |
| n |  | 10577 | 5274 | 4445 | 858 |  |
| Male (%) |  | 7851 (74.2) | 4257 (80.7) | 3118 (70.1) | 476 (55.5) | <0.001 |
| Age, y |  | 61.40 [54.20, 67.50] | 57.00 [50.90, 63.20] | 64.20 [58.30, 69.90] | 69.60 [63.60, 75.47] | <0.001 |
| BMI, kg/m2 |  | 26.01 [24.13, 28.07] | 26.12 [24.22, 28.30] | 25.95 [24.03, 28.01] | 25.95 [23.85, 27.88] | <0.001 |
| **Clinical presentation** | | | | | | |
| ACS (%) |  | 6827 (64.5) | 3387 (64.2) | 2841 (63.9) | 599 (69.8) | 0.003 |
| Atrial fibrillation (%) |  | 417 (3.9) | 111 (2.1) | 226 (5.1) | 80 (9.3) | <0.001 |
| pre-PCI (%) |  | 3218 (30.4) | 1503 (28.5) | 1417 (31.9) | 298 (34.7) | <0.001 |
| preCABG (%) |  | 292 (2.8) | 103 (2.0) | 142 (3.2) | 47 (5.5) | <0.001 |
| Dyslipidemia (%) |  | 8422 (79.6) | 4120 (78.1) | 3616 (81.3) | 686 (80.0) | <0.001 |
| HTN (%) |  | 7271 (68.7) | 3306 (62.7) | 3251 (73.1) | 714 (83.2) | <0.001 |
| Family History CAD (%) |  | 1240 (11.7) | 693 (13.1) | 467 (10.5) | 80 (9.3) | <0.001 |
| Smoking (%) |  | 6728 (63.6) | 3647 (69.2) | 2656 (59.8) | 425 (49.5) | <0.001 |
| **Laboratory data** | | | | | | |
| LVEF, % |  | 62.00 [60.00, 65.00] | 62.00 [60.00, 65.00] | 62.00 [60.00, 65.00] | 60.00 [55.00, 65.00] | <0.001 |
| BNP, pg/ml |  | 111.20 [46.40, 341.30] | 87.10 [37.92, 247.40] | 125.10 [51.40, 360.10] | 388.85 [122.55, 1477.00] | <0.001 |
| ET, pmol/L |  | 0.25 [0.19, 0.38] | 0.23 [0.17, 0.34] | 0.27 [0.20, 0.39] | 0.38 [0.25, 0.53] | <0.001 |
| Glu, mmol/L |  | 7.57 [6.40, 9.43] | 7.58 [6.42, 9.43] | 7.51 [6.36, 9.31] | 8.06 [6.60, 10.23] | <0.001 |
| HbA1C, % |  | 7.10 [6.50, 8.10] | 7.00 [6.40, 8.10] | 7.00 [6.50, 8.10] | 7.20 [6.50, 8.17] | 0.006 |
| TC, mmol/L |  | 3.85 [3.25, 4.59] | 3.83 [3.22, 4.57] | 3.87 [3.27, 4.60] | 4.00 [3.35, 4.80] | <0.001 |
| TG, mmol/L |  | 1.52 [1.14, 2.14] | 1.49 [1.11, 2.10] | 1.52 [1.15, 2.15] | 1.70 [1.27, 2.33] | <0.001 |
| LDL, mmol/L |  | 2.25 [1.76, 2.88] | 2.23 [1.75, 2.87] | 2.25 [1.76, 2.87] | 2.33 [1.81, 3.00] | 0.006 |
| HDL, mmol/L |  | 1.04 [0.88, 1.23] | 1.02 [0.86, 1.21] | 1.06 [0.89, 1.25] | 1.05 [0.87, 1.26] | <0.001 |
| UA, mmol/L |  | 334.38 [279.55, 394.22] | 317.20 [268.05, 374.06] | 342.93 [286.94, 403.15] | 399.71 [340.36, 473.80] | <0.001 |
| Ka, mmol/L |  | 4.11 [3.85, 4.37] | 4.04 [3.79, 4.29] | 4.16 [3.89, 4.42] | 4.31 [4.01, 4.60] | <0.001 |
| Na, mmol/L |  | 139.60 [137.93, 141.17] | 139.50 [137.80, 141.00] | 139.70 [138.10, 141.34] | 139.30 [137.48, 141.00] | <0.001 |
| CRP, mmol/L |  | 2.99 [1.95, 5.58] | 2.92 [1.89, 5.33] | 2.93 [1.97, 5.47] | 3.87 [2.39, 8.09] | <0.001 |
| HBG, g/dL |  | 4.74 [4.36, 5.12] | 4.78 [4.43, 5.14] | 4.75 [4.35, 5.12] | 4.42 [4.01, 4.85] | <0.001 |
| PLT, K/uL |  | 221.00 [184.00, 262.00] | 221.00 [184.00, 261.00] | 220.00 [185.00, 262.00] | 224.00 [185.00, 267.75] | 0.18 |
| WBC, K/uL |  | 6.85 [5.75, 8.21] | 6.88 [5.74, 8.33] | 6.75 [5.73, 7.99] | 7.26 [6.04, 8.65] | <0.001 |
| ALT, mmol/L |  | 24.00 [17.00, 37.00] | 26.00 [18.00, 39.00] | 23.00 [16.00, 34.00] | 21.00 [14.00, 31.00] | <0.001 |
| AST, mmol/L |  | 22.00 [18.00, 29.00] | 22.00 [18.00, 29.00] | 22.00 [18.00, 28.00] | 22.00 [18.00, 29.00] | 0.764 |
| BUN, mmol/L |  | 5.40 [4.45, 6.50] | 4.95 [4.17, 5.85] | 5.70 [4.78, 6.73] | 7.92 [6.51, 9.43] | <0.001 |
| eGFR, mL/min/1.73m2 |  | 89.93 [76.38, 99.76] | 99.83 [95.45, 105.00] | 78.89 [71.71, 84.81] | 52.78 [46.11, 56.59] | <0.001 |
| **Angiographic and PCI data** | | | | | | |
| Heavily calcified (%) |  | 406 (3.8) | 172 (3.3) | 184 (4.1) | 50 (5.8) | 0.001 |
| Syntax |  | 12.00 [7.00, 20.00] | 12.00 [7.00, 19.00] | 12.00 [7.00, 20.00] | 14.50 [8.00, 21.88] | <0.001 |
| Anomalous origin coronary (%) | | 78 (0.7) | 35 (0.7) | 35 (0.8) | 8 (0.9) | 0.61 |
| Mono vessel (%) |  | 1643 (15.5) | 897 (17.0) | 653 (14.7) | 93 (10.8) | <0.001 |
| Dual vessel (%) |  | 3294 (31.1) | 1730 (32.8) | 1306 (29.4) | 258 (30.1) | 0.001 |
| Triple vessel (%) |  | 5626 (53.2) | 2642 (50.1) | 2477 (55.7) | 507 (59.1) | <0.001 |
| LM disease (%) |  | 1127 (10.7) | 502 (9.5) | 515 (11.6) | 110 (12.8) | <0.001 |
| MLD, mm |  | 0.30 [0.12, 0.56] | 0.30 [0.04, 0.56] | 0.30 [0.12, 0.56] | 0.28 [0.03, 0.50] | 0.003 |
| DS, mm |  | 90.00 [80.00, 95.00] | 90.00 [80.00, 99.00] | 90.00 [80.00, 95.00] | 90.00 [80.00, 99.00] | 0.004 |
| TIMI (%) | 0 | 2057 (19.4) | 1052 (19.9) | 815 (18.3) | 190 (22.1) | 0.084 |
|  | 1 | 397 (3.8) | 209 (4.0) | 160 (3.6) | 28 (3.3) |  |
|  | 2 | 1099 (10.4) | 548 (10.4) | 457 (10.3) | 94 (11.0) |  |
|  | 3 | 7024 (66.4) | 3465 (65.7) | 3013 (67.8) | 546 (63.6) |  |
| B2_C_lesion (%) |  | 7837 (74.1) | 3943 (74.8) | 3254 (73.2) | 640 (74.6) | 0.205 |
| Diffused Range (%) |  | 6267 (59.3) | 3134 (59.4) | 2630 (59.2) | 503 (58.6) | 0.897 |
| Extremely Tortuosity (%) |  | 286 (2.7) | 142 (2.7) | 119 (2.7) | 25 (2.9) | 0.924 |
| Angulated (%) |  | 3388 (32.0) | 1677 (31.8) | 1451 (32.6) | 260 (30.3) | 0.354 |
| Irregular (%) |  | 8124 (76.8) | 3990 (75.7) | 3460 (77.8) | 674 (78.6) | 0.018 |
| CTO (%) |  | 1041 (9.8) | 499 (9.5) | 445 (10.0) | 97 (11.3) | 0.215 |
| Ostial (%) |  | 1299 (12.3) | 627 (11.9) | 548 (12.3) | 124 (14.5) | 0.104 |
| Bifurcation (%) |  | 1948 (18.4) | 1017 (19.3) | 784 (17.6) | 147 (17.1) | 0.068 |
| Stent (%) |  | 9523 (90.0) | 4768 (90.4) | 4002 (90.0) | 753 (87.8) | 0.056 |
| **Medications at discharge** | | | | | | |
| ARB/ACEI (%) |  | 2890 (27.3) | 1331 (25.2) | 1276 (28.7) | 283 (33.0) | <0.001 |
| CCB (%) |  | 4522 (42.8) | 2015 (38.2) | 2040 (45.9) | 467 (54.4) | <0.001 |
| NIT (%) |  | 10218 (96.6) | 5082 (96.4) | 4318 (97.1) | 818 (95.3) | 0.011 |
| beta (%) |  | 9631 (91.1) | 4845 (91.9) | 4008 (90.2) | 778 (90.7) | 0.013 |
| statin (%) |  | 10461 (98.9) | 5218 (98.9) | 4396 (98.9) | 847 (98.7) | 0.847 |
| Aspirin (%) |  | 10531 (99.6) | 5256 (99.7) | 4425 (99.6) | 850 (99.1) | 0.05 |
| Clopidogrel (%) |  | 10424 (98.6) | 5193 (98.5) | 4384 (98.6) | 847 (98.7) | 0.73 |
| Ticagrelor (%) |  | 2087 (19.7) | 1170 (22.2) | 777 (17.5) | 140 (16.3) | <0.001 |
| LMWH (%) |  | 7115 (67.3) | 3641 (69.0) | 2912 (65.5) | 562 (65.5) | 0.001 |
| Oral hypoglycaemics (%) |  | 5146 (48.7) | 2672 (50.7) | 2086 (46.9) | 388 (45.2) | <0.001 |
| Insulin (%) |  | 1764 (16.7) | 737 (14.0) | 813 (18.3) | 214 (24.9) | <0.001 |
| DM Dietary Control (%) |  | 780 (7.4) | 405 (7.7) | 334 (7.5) | 41 (4.8) | 0.01 |
|  |  |  |  |  |  |  |
| ET-1; Endothelin-1; BMI, body mass index; WBC, white blood cell; HBG, hemoglobin; PLT, platelet; eGFR, estimated glomerular filtration rate; ACS, acute coronary syndrome; DM, diabetes mellitus； PCI, percutaneous coronary intervention; TG, triglycerides; TC, total cholesterol; LDL-C, low-density lipoprotein cholesterol; HDL-C, high-density lipoprotein cholesterol; MLD, minimal lumen diameter; TIMI, Thrombolysis In Myocardial Infarction; LM, left main coronary artery; SYNTAX, synergy between percutaneous coronary intervention with TAXUS and cardiac surgery; BNP, brain natriuretic peptide; CCB, calcium channel blockers; ARB/ACEI, angiotensin II receptor blockers/angiotensin-converting enzyme inhibitors; LMWH, low molecular weight heparin; CTO, chronic total occlusion. | | | | | | |

| **Supplementary Table 4 Univariate and multivariate Cox regression analyses for various adverse clinical outcomes risk related to ET-1 levels in CAD patients with DM.** | | | |
| --- | --- | --- | --- |
| **MACE** | **events** | **robust Model** | **adjusted Model** |
| Low ET-1 | 405 | reference | reference |
| Medium ET-1 | 416 | 1.104(0.963-1.266) | 1.030(0.897-1.183) |
| High ET-1 | 542 | **1.492(1.311-1.698) ^a^** | **1.279(1.118-1.464) ^a^** |
| **All-cause death** |  |  |  |
| Low ET-1 | 53 | reference | reference |
| Medium ET-1 | 78 | **1.556(1.098-2.206) ^a^** | 1.350(0.951-1.918) |
| High ET-1 | 173 | **3.531(2.595-4.805) ^a^** | **2.188(1.585-3.022) ^a^** |
| **CV-cause death** |  |  |  |
| Low ET-1 | 12 | reference | reference |
| Medium ET-1 | 40 | **3.519(1.846-6.709) ^a^** | **3.161(1.649-6.058) ^a^** |
| High ET-1 | 72 | **6.508(3.529-11.990) ^a^** | **3.824(2.028-7.210) ^a^** |
| **Nonfatal myocardial infarction** | |  |  |
| Low ET-1 | 64 | reference | reference |
| Medium ET-1 | 92 | **1.606(1.167-2.212) ^a^** | **1.436(1.038-1.987) ^a^** |
| High ET-1 | 126 | **2.3781(1.757-3.219) ^a^** | **1.970(1.436-2.702) ^a^** |
| **Stroke** |  |  |  |
| Low ET-1 | 17 | reference | reference |
| Medium ET-1 | 19 | 1.177(0.612-2.265) | 1.105(0.572-2.135) |
| High ET-1 | 28 | 1.767(0.967-3.229) | 1.491(0.797-2.791) |
| **Revascularization** |  |  |  |
| Low ET-1 | 334 | reference | reference |
| Medium ET-1 | 315 | 1.007(0.863-1.175) | 1.028(0.895-1.182) |
| High ET-1 | 336 | 1.111(0.955-1.294) | 1.066(0.911-1.249) |
|  |  |  |  |
| ET-1; Endothelin-1; MACE, major adverse cardiovascular events; CV, cardiovascular. | | | |
| **^a^** P<0.05 |  |  |  |
| Adjusted Model adjusted for age, sex, body mass index (BMI), health status (acute coronary disease, smoking status, hypertension, dyslipidemia, family history of CAD), brain natriuretic peptide levels, medications at discharge (statins, aspirin, clopidogrel, ticagrelor, angiotensin II receptor blockers/angiotensin-converting enzyme inhibitors, beta-blockers, and calcium channel blockers), and angiographic characteristics (e.g., heavily calcified lesions, Syntax score, lesion length, chronic total occlusion, bifurcation, stent usage). | | | |

| **Supplementary Table 5 Risk of all-cause death according to diabetic status and ET-1 level from multivariate Cox regression analyses in CAD patients with impaired kidney function.** | | | | |
| --- | --- | --- | --- | --- |
| ET-1 | Events | Subjects | robust Model | adjusted Model |
| non-DM |  |  |  |  |
| Low ET-1 | 7 | 207 | reference | reference |
| Medium ET-1 | 5 | 172 | 0.849(0.269-2.678) | 0.695(0.218-2.211) |
| High ET-1 | 13 | 107 | **3.822(1.524-9.585)** ^a^ | **2.593(1.008-6.664)** ^a^ |
| DM |  |  |  |  |
| Low ET-1 | 9 | 258 | 1.081(0.403-2.904) | 1.019(0.374-2.781) |
| Medium ET-1 | 31 | 277 | **3.641(1.603-8.270)** ^a^ | **3.153(1.372-7.249)** ^a^ |
| High ET-1 | 64 | 323 | **6.378(2.922-13.921)** ^a^ | **3.933(1.750-8.838)** ^a^ |
|  |  |  |  |  |
| ET-1; Endothelin-1; DM, diabetes mellitus. | | |  |  |
| ^a^ P<0.05 |  |  |  |  |
| Adjusted Model adjusted for age, sex, body mass index (BMI), health status (acute coronary disease, smoking status, hypertension, dyslipidemia, family history of CAD), brain natriuretic peptide levels, medications at discharge (statins, aspirin, clopidogrel, ticagrelor, angiotensin II receptor blockers/angiotensin-converting enzyme inhibitors, beta-blockers, and calcium channel blockers), and angiographic characteristics (e.g., heavily calcified lesions, Syntax score, lesion length, chronic total occlusion, bifurcation, stent usage). | | | | |

| **Supplementary Table 6 Risk of CV-cause death according to diabetic status and ET-1 level from multivariate Cox regression analyses in CAD patients with impaired kidney function.** | | | | |
| --- | --- | --- | --- | --- |
| ET-1 | Events | Subjects | robust Model | adjusted Model |
| non-DM |  |  |  |  |
| Low ET-1 | 1 | 207 | reference | reference |
| Medium ET-1 | 2 | 172 | 2.317(0.210-25.560) | 2.004(0.178-22.544) |
| High ET-1 | 5 | 107 | **10.154(1.186-86.958) ^a^** | 8.213(0.936-72.054) |
| DM |  |  |  |  |
| Low ET-1 | 2 | 258 | 1.681(0.152-18.544) | 1.745(0.159-19.534) |
| Medium ET-1 | 11 | 277 | **9.044(1.167-70.065) ^a^** | **8.851(1.125-69.642) ^a^** |
| High ET-1 | 35 | 323 | **24.233(3.319-176.916) ^a^** | **15.436(2.052-116.139) ^a^** |
|  |  |  |  |  |
| ET-1; Endothelin-1; CV, cardiovascular; DM, diabetes mellitus. | | | |  |
| **^a^** P<0.05 |  |  |  |  |
| Adjusted Model adjusted for age, sex, body mass index (BMI), health status (acute coronary disease, smoking status, hypertension, dyslipidemia, family history of CAD), brain natriuretic peptide levels, medications at discharge (statins, aspirin, clopidogrel, ticagrelor, angiotensin II receptor blockers/angiotensin-converting enzyme inhibitors, beta-blockers, and calcium channel blockers), and angiographic characteristics (e.g., heavily calcified lesions, Syntax score, lesion length, chronic total occlusion, bifurcation, stent usage). | | | | |

| **Supplementary Table 7 Risk of MI according to diabetic status and ET-1 level from multivariate Cox regression analyses in CAD patients with impaired kidney function.** | | | | |
| --- | --- | --- | --- | --- |
| ET-1 | Events | Subjects | robust Model | adjusted Model |
| non-DM |  |  |  |  |
| Low ET-1 | 4 | 207 | reference | reference |
| Medium ET-1 | 1 | 172 | 0.272(0.030-2.451) | 0.182(0.016-2.043) |
| High ET-1 | 5 | 107 | 2.815(0.754-10.517) | 2.739(0.681-11.014) |
| DM |  |  |  |  |
| Low ET-1 | 7 | 258 | 1.576(0.460-5.397) | 1.665(0.464-5.983) |
| Medium ET-1 | 11 | 277 | 2.492(0.791-7.851) | 2.292(0.696-7.549) |
| High ET-1 | 23 | 323 | **3.636(1.242-10.643) ^a^** | 2.956(0.939-9.307) |
|  |  |  |  |  |
| ET-1; Endothelin-1; MI, myocardial infarction; DM, diabetes mellitus. | | | |  |
| **^a^** P<0.05 |  |  |  |  |
| Adjusted Model adjusted for age, sex, body mass index (BMI), health status (acute coronary disease, smoking status, hypertension, dyslipidemia, family history of CAD), brain natriuretic peptide levels, medications at discharge (statins, aspirin, clopidogrel, ticagrelor, angiotensin II receptor blockers/angiotensin-converting enzyme inhibitors, beta-blockers, and calcium channel blockers), and angiographic characteristics (e.g., heavily calcified lesions, Syntax score, lesion length, chronic total occlusion, bifurcation, stent usage). | | | | |
